# Enhancement of LTE and NR systems through efficient physical cell identity allocation

**DOI:** 10.1038/s41598-026-36608-w

**Published:** 2026-02-09

**Authors:** Samar I. Farghaly, Hala M. Khayal, Ibrahim M. Algohary, Mahmoud A. Eissa, Marwan H. Elsheikh, Passant E. Alnashar, Rana S. Alfakhrany, Roqaya S. Alkhuoty, Ahmed S. Abo-Alsayed

**Affiliations:** Electronics and Electrical Communications Engineering Department, Faculty of Engineering, Tanta University, Tanta, Egypt

**Keywords:** Engineering, Mathematics and computing

## Abstract

As mobile communication systems continue to evolve to support higher data rates, ultra-low latency, and greater reliability, the effective management of Physical Cell Identities (PCI) becomes increasingly important. The pool of available identifiers is limited—504 in fourth-generation networks and 1008 in fifth-generation networks—so reuse of these identities is unavoidable. Improper reuse can lead to collisions and confusion, resulting in interference, failed handovers, and reduced network throughput. This work addresses these challenges by exploring advanced automated techniques for optimizing PCI. We model the network as a graph, where each cell is represented as a vertex and interference relationships between cells are represented as edges. Using this approach, we implement graph coloring algorithms, including a maximum degree first coloring method, to efficiently assign identifiers and minimize conflicts. We also introduce clustering-based methods, such as Fuzzy hierarchical clustering, to dynamically group cells based on real-time traffic patterns, enabling adaptive management in dense networks. Beyond these deterministic methods, we evaluate metaheuristic approaches, including Biased Random-Key Genetic Algorithms (BRKG), Integer Linear Programming (ILP), and constructive heuristics based on DSATUR. Where they are particularly effective in navigating complex solution spaces to generate near-optimal PCI plans. Our simulation results demonstrate that these modern techniques significantly outperform traditional strategies, reducing identity conflicts by up to 80% and improving key performance metrics such as signal quality, handover success rates, and overall network throughput.

## Introduction

 Mobile networks are rapidly advancing to meet growing demands for higher data rates, ultra-low latency, and highly reliable connectivity. The evolution from 4G LTE to 5G New Radio (NR) marks a major transformation, introducing powerful capabilities alongside increased network complexity. This evolution introduces significant complexity in network design, deployment, and optimization. 5G networks rely on dense topologies with small cells, utilize new high-frequency bands in the millimeter-wave (mmWave) spectrum, and incorporate a wide range of configurable parameters. While mmWave bands above 28 GHz provide large bandwidths essential for high data rates, they also present challenges such as increased propagation losses, susceptibility to atmospheric absorption, and blockage by physical objects. Regulatory bodies, such as the FCC, have increased the minimum bandwidth for mmWave to 400 MHz, compared to LTE’s 100 MHz and it is varies by allocation, further necessitating high-gain antennas to overcome these losses. Network engineers face the critical task of ensuring efficient, reliable, and seamless network performance under these complex conditions. One of the foundational elements of network operation that directly impacts performance is Physical Cell Identity (PCI) planning. Among the most critical tasks in radio network engineering is the planning and allocation of the PCI^1–12,12^ the key physical-layer identifier that enables User Equipment (UE) to recognize cells, synchronize with the network, and execute handovers. However, the PCI pool is limited to just 504 identities in LTE and 1008 in 5G, making reuse inevitable across large deployments. Poorly planned reuse leads to PCI collisions and confusion, causing co-channel interference, dropped connections, failed handovers, and degraded user experience. Consequently, the PCI is a fundamental parameter in both 4G LTE and 5G networks^13,15–17,17^, influencing Quality of Service (QoS), user experience, and network efficiency. PCI enables UE to identify individual cells, synchronize with the network, and perform handovers. It is derived from the Primary Synchronization Signal (PSS) and Secondary Synchronization Signal (SSS), forming the basis for network operations such as:

- Initial Network Access: When a UE powers on or enters a new area, it scans for synchronization signals. The PCI allows the UE to identify the cell and synchronize timing, which is essential for establishing any communication^18,20,20^.
- Seamless Handover: As the UE moves, it measures signals from neighboring cells, reports the data to the serving cell, and enables intelligent handover decisions. Unique PCI assignments are critical for uninterrupted service during mobility^21^.

In LTE and 5G NR, each cell is identified by a Cell ID organized into groups and sectors. The group ranges from 0 to 335, and the sector ID ranges from 0 to 2. The UE determines sector ID from the PSS and group ID from the SSS, then computes the Cell-ID. While 5G NR retains this structure, it introduces new synchronization sequences to support scalable deployments across diverse frequency bands. Limited PCI pools (504 in LTE, 1008 in 5G) make reuse unavoidable. Poorly planned reuse causes PCI collisions and confusions, resulting in call drops, handover failures, inter-cell interference, and reduced throughput. These challenges are due to massive MIMO and beamforming where highly directional beams change interference patterns dynamically, making static PCI plans insufficient. In addition, TDD operation which Shared uplink and downlink frequencies require precise synchronization to avoid cross-link interference. Finally, modulo conflicts, Certain PCI pairings (mod3, mod6, mod30) create structured interference affecting SSS, PSS, uplink reference signals, and PCFICH channels. For example, PCI-mod3 planning ensures sector orthogonality in 3-sector sites, while PCI-mod6 is critical in mixed MIMO/SISO deployments. Mod30 patterns may affect uplink decoding, and specific PCFICH collisions occur every 25–50 PCIs depending on channel bandwidth. These issues are especially critical in dense urban deployments, such as Egypt’s 5G rollout at 2600 MHz TDD, where synchronization and interference management are vital. So, Modern PCI planning requires automated, intelligent allocation strategies such as^22,24,24^:

1. **Graph-Based Coloring Algorithms (e.g.**,** MDFC)**: Cells are modeled as graph vertices, and potential conflicts as edges. PCI assignment becomes a graph coloring problem, with algorithms like DSATUR prioritizing high-degree nodes to resolve critical conflicts first^25^.
2. **Clustering Techniques (e.g.**,** Fuzzy Hierarchical Clustering - FHC)**: Cells are grouped by proximity, traffic, or other metrics. Clusters enable optimized PCI reuse patterns based on local conditions^26,27^.
3. **Metaheuristic Algorithms (e.g.**,** BRKGA**,** Simulated Annealing(SA))**: Nature-inspired methods explore large, complex search spaces to find near-optimal PCI assignments, particularly for problems where exact solutions are computationally infeasible^28,30,31,31^.

These advanced strategies enable conflict-free PCI allocation, improve interference management, and ensure seamless mobility in dynamic 5G environments^32^.

This paper delivers a structured analysis of this challenge, moving from the fundamentals of PCI planning to the design and evaluation of advanced automated optimization strategies. By modeling the cellular network as a graph and applying a range of techniques from exact methods like Integer Linear Programming to heuristics such as graph coloring and metaheuristic algorithms like Genetic Algorithms and Simulated Annealing aim to generate intelligent, conflict-free PCI allocation schemes. These methods are not only proposed but rigorously validated through a high-fidelity simulation framework built in Network Simulator 3 (NS-3), offering clear evidence of their effectiveness in improving real-world network performance.

### Problem statement

As established, in dense 5G networks, PCI allocation is not merely a technical detail but a crucial factor that directly impacts several core network functionalities:

- Seamless UE Synchronization: The ability of a UE to quickly and accurately synchronize with a cell’s timing and frame structure is the very first step in establishing a connection. Incorrect PCI assignments can disrupt this fundamental process, leading to delayed network access or outright failures^33^.
- Efficient Handover Operations: As UEs move between cells, handovers must occur smoothly and without interruption to maintain continuous service. Ambiguous PCI information can cause handovers to fail, leading to call drops and data session interruptions.
- Minimal Interference and Signal Degradation: PCIs are used to generate unique reference signals that help UEs distinguish cells. When PCIs are poorly planned, these signals can collide or be confused, leading to significant inter-cell interference that degrades signal quality and reduces achievable data rates for all users^34^.

The fundamental challenge stems from the limited pool of available PCI values: 504 total for 4G LTE and 1008 for 5G. This inherent scarcity means that PCI reuse is inevitable across a large network deployment.

### Research gap

Traditional methods for PCI allocation have proven increasingly inadequate in the face of the growing complexity and demands of modern mobile networks, particularly with the advent of 5G and its dense heterogeneous deployments. These conventional approaches suffer from inherent limitations that make them ill-suited for optimizing network performance in dynamic and interference-sensitive environments^18,20–37,36^.

Historically, PCI allocation is often relied on manual assignments by network engineers. This approach is inherently prone to significant inefficiencies and human error, especially in large-scale deployments. The sheer volume of cells and the intricate web of inter-cell relationships make it virtually impossible for human planners to account for all potential conflicts and optimize for global network performance^13,1^. This can lead to oversight, slow deployment, and frequent reconfigurations post-deployment as issues emerge. In some simplified scenarios or as a baseline, PCIs might be allocated randomly. However, this method consistently leads to high levels of interference and necessitates frequent, costly reconfigurations. Random allocation disregards critical factors such as geographical proximity, traffic load, and antenna patterns, thereby maximizing the probability of PCI collisions and confusions from the outset. While ANR mechanisms automatically detect and establish neighbor relationships, basic ANR-based PCI assignment schemes often lack the advanced intelligence required for proactive conflict prevention and global optimization. They typically react to detected conflicts rather than predicting and preventing them, and they may not adapt well to the highly dynamic nature of network traffic and changing radio conditions. Such basic ANR systems are often insufficient in maintaining optimal performance in the face of rapid cell densification and evolving interference landscapes^37,42–44,41^. The collective shortcomings of these traditional methods highlight a significant research gap: there is a critical need for intelligent, automated, and adaptive PCI allocation strategies that can proactively minimize interference, optimize resource utilization, and ensure seamless connectivity in complex 4G and 5G network environments. The subsequent sections explore advanced methodologies designed to address this pressing challenge^37,42,43,40^.

### Contribution

This paper addresses a key challenge in modern wireless communications: efficient PCI planning for LTE and NR networks. As mobile networks evolve to support data-intensive applications from HD video streaming to IoT and autonomous systems network densification places increasing pressure on limited resources, particularly PCIs^41^.

PCIs act as unique physical-layer identifiers, enabling UEs to distinguish cells, synchronize, and perform seamless handovers. LTE provides only 504 unique PCIs and 5G NR 1008, necessitating careful reuse in dense metropolitan deployments^39^. Poor PCI management can cause PCI collisions, where adjacent cells share the same PCI, leading to synchronization failures, and PCI confusion, where multiple neighbors share a PCI, resulting in failed handovers. Inefficient PCI allocation degrades SINR, reduces throughput, and lowers overall network performance. The transition to 5G, with Massive MIMO, dynamic beamforming, and TDD, further complicates interference, rendering traditional neighbor lists insufficient. This underscores the need for intelligent, automated, and adaptive PCI planning strategies^41,46–51,48^.

The main contributions of this paper are summarized as follows:

- Unlike existing PCI allocation studies that rely on fixed or full neighbor lists, this work introduces a top-neighbor concept, where only the most influential neighboring cells are considered during optimization. This strategy better reflects realistic interference dominance, reduces unnecessary constraints, and significantly improves scalability in dense LTE/NR deployments. To the best of our knowledge, this concept has not been explicitly incorporated into PCI planning formulations in prior literature.
- While DSATUR itself is a classical graph-coloring algorithm, this paper proposes an enhanced execution strategy, where DSATUR is executed multiple times with different node orderings. The resulting solutions are then evaluated and compared, and the best-performing PCI assignment is selected.
- This modification improves solution robustness and quality without increasing algorithmic complexity and represents a practical enhancement beyond standard single-run DSATUR implementations.
- This work presents, to the authors’ knowledge, the first application of a Multi-Population Biased Random-Key Genetic Algorithm (MP-BRKGA) to the PCI planning problem.
- The proposed formulation adapts BRKGA to handle PCI collision and confusion constraints, demonstrating that evolutionary optimization can effectively explore large PCI search spaces beyond what traditional graph-based heuristics can achieve.
- To overcome the scalability limitations of classical ILP formulations, this paper introduces a clustering-assisted ILP approach, where the network is first partitioned into smaller subgraphs before optimization.
- This hybrid strategy allows ILP to produce feasible solutions for larger and more highly connected networks than previously possible, bridging the gap between exact optimization and practical deployment.
- The paper introduces a custom SINR penalty model integrated into NS-3 to realistically evaluate the impact of PCI collisions and confusion on system performance. Unlike prior works that rely solely on abstract graph metrics, this study links PCI planning decisions to network-level SINR degradation, enabling performance assessment in terms of interference, throughput, and handover reliability. The full configuration parameters and equations are provided to ensure reproducibility.
- The study provides a fair and systematic comparison of DSATUR, MP-BRKGA, and ILP-based methods under unified network models, identical constraints, and consistent evaluation metrics.
- Both computational scalability (runtime and feasibility) and network-level performance are analyzed, offering practical insights into algorithm suitability under different deployment scenarios.

## Problem formulation

In 4G LTE networks, Radio Resource Allocation (RRA) occurs on the downlink (DL) from eNodeBs to UE. The eNodeB gathers information about channel conditions through Channel State Information (CSI) and considers application requirements based on QoS specifications. Resource allocation is performed using 10 ms radio frames arranged in a time-frequency grid. Each frame is divided into 1 ms subframes, which are further split into 0.5 ms slots. Within each slot, a user request is assigned to 7 OFDM symbols, and a Resource Block (RB) consists of 12 subcarriers, each 180 kHz wide. RRA algorithms are typically classified as channel-aware, which adapt to varying channel conditions, or QoS-aware, which prioritize allocation according to application QoS requirements^49^. Theoretically, the minimum allocatable resource to a user is a single Resource Element (RE). In practice, however, each user is typically assigned 2 RBs, with each RB consisting of 12 subcarriers in one subframe. Not all allocated REs carries user data; some are reserved for Reference Signals (RSs), which are transmitted in specific REs within the Physical Resource Blocks (PRBs)^50^. In LTE, these reference signals play a critical role in network operations. Their primary functions such as channel estimation, synchronization and cell Identification, channel state information (CSI) feedback, interference mitigation and handover support. In a MIMO system, when one of the transmit antennas is sending its reference signal, the other antenna temporarily halts transmission. This coordination allows the receiver to accurately perform channel estimation, distinguishing the individual channel characteristics of each transmit antenna without interference from the others.

From a modeling perspective, the PCI allocation problem is formulated as the minimization of a weighted interference cost function, expressed as

1 $$\:\underset{p}{\mathrm{m}\mathrm{i}\mathrm{n}}{\hspace{0.25em}\hspace{0.05em}}\sum\:_{i=1}^{N}\sum\:_{j\in\:{\mathcal{N}}_{i}}{w}_{ij}{\hspace{0.17em}}\mathbb{I}\left(\mathrm{PSS}\right({p}_{i})=\mathrm{PSS}({p}_{j}\left)\right)$$

where $\:{p}_{i}$denotes the PCI assigned to cell $\:i$, $\:{\mathcal{N}}_{i}$is the RSRP-based neighborhood set obtained from ns-3 simulations, $\:{w}_{ij}$represents the measured interference weight between cells $\:i$and $\:j$, and $\:\mathbb{I}(\cdot\:)$is the indicator function. Optimization is subject to practical PCI constraints, such as

$$\:\left({p}_{i}{\hspace{0.05em}}mod{\hspace{0.05em}}3\right)\ne\:\left({p}_{j}{\hspace{0.05em}}mod{\hspace{0.05em}}3\right),\left({p}_{i}{\hspace{0.05em}}mod{\hspace{0.05em}}6\right)\ne\:\left({p}_{j}{\hspace{0.05em}}mod{\hspace{0.05em}}6\right),\left({p}_{i}{\hspace{0.05em}}mod{\hspace{0.05em}}30\right)\ne\:\left({p}_{j}{\hspace{0.05em}}mod{\hspace{0.05em}}30\right),$$.

for all conflicting neighboring cells $\:j\in\:{\mathcal{N}}_{i}$, which explicitly model PSS/SSS reuse rules in real LTE/NR deployments.

Due to the NP-hard nature of the underlying PCI assignment problem and the heuristic or metaheuristic nature of several evaluated algorithms, deriving tight analytical performance guarantees is not tractable and is beyond the scope of this work. Instead, the proposed framework relies on controlled, repeatable simulations to evaluate convergence behavior, solution quality, and robustness under realistic radio conditions. Importantly, the results reveal non-trivial and practically relevant behaviors, such as sensitivity to RSRP thresholds, user density, and mobility patterns, as well as differences in stability and solution consistency across algorithms. These effects are not captured by abstract theoretical models yet are critical for real-world PCI planning and optimization. The following subsections describe the problems in detail.

### Synchronization signals

In 5G NR, the Primary and Secondary Synchronization Signals (PSS and SSS) help mobile devices (UEs) detect which cell they are connected to and align with the network’s timing. Each cell is assigned a unique ID from a pool of 1008, organized into 336 groups. The UE figures out the **sector ID** from the PSS and the **group ID** from the SSS, then combines them to get the full cell ID^49^:

2 $$\:{N}_{\mathrm{ID}}^{\mathrm{cell}}=3{N}_{\mathrm{ID}}^{\left(1\right)}+{N}_{\mathrm{ID}}^{\left(2\right)}$$

While LTE and NR share the same general approach, 5G NR uses longer sequences and slightly different mappings to handle its flexible deployment scenarios. In our simulations, we use the standard PSS/SSS placement from 3GPP to realistically model interference and SINR for PCI planning. The detailed mathematical derivations of these sequences are not included, as they are well-established in 3GPP TS 36.211 (LTE) and TS 38.211 (NR) and do not affect the focus of this work.

### How the UE uses PSS and SSS for synchronization

- Detects the PSS and Determines slot timing and identifies one of three possible cell identities.
- Detects the SSS and Determines the full Physical Cell ID (0–1007) and frame timing.
- Uses PCI for decoding the Physical Broadcast Channel (PBCH) and completing initial access to the LTE or NR cell.

Primary Synchronization Signals (PSS) and Secondary Synchronization Signals (SSS) are fundamental components for synchronization in wireless systems. Figure 3.6 illustrates how the locations of the PSS are identified within the subframes (SFs), where we observe it is positioned at the sixth location in both subframe SF0 and subframe SF5. Similarly, Fig. 3.7 shows the SSS locations within the subframes, also found at the sixth position in both subframe SF0 and subframe SF5. This standardized arrangement highlights the importance of these signals in successfully completing the synchronization process.

### Key PCI planning rules

The Physical Cell Identity (PCI) is derived from the Secondary Synchronization Signal (SSS) and the Primary Synchronization Signal (PSS) using the formula^52^:

3 $${\text{PCI = 3 }} \times SSS + PSS\;$$

where PSS = 0, 1, 2. For 4G LTE: SSS ranges from 0 to 167, giving a total of 504 PCIs (0–503) and for 5G NR: SSS ranges from 0 to 335, resulting in 1008 PCIs (0–1007).

### PCI planning rules and guidelines

**Fig. 1:**
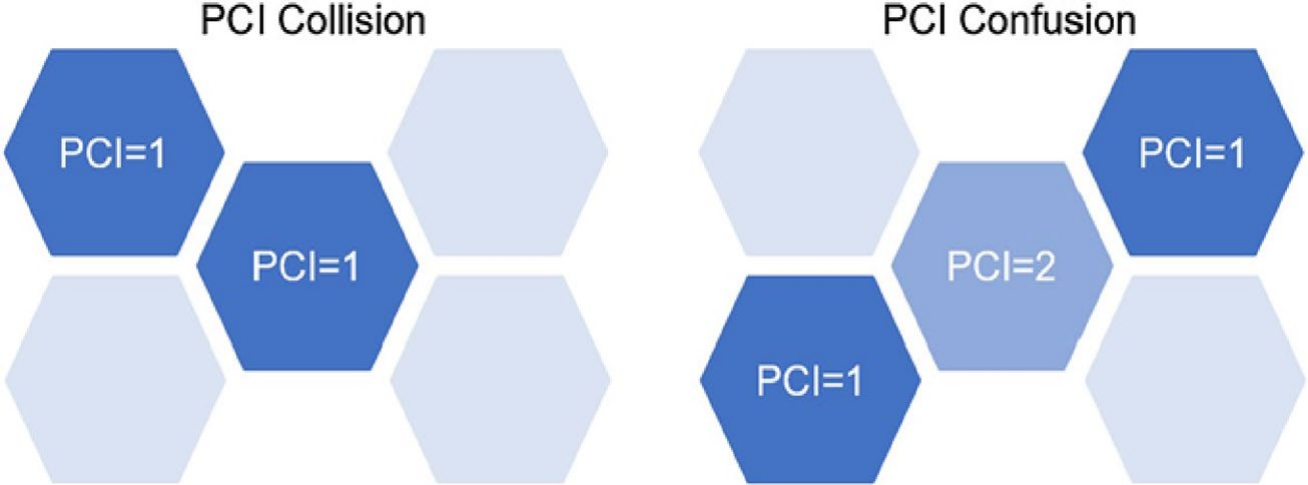
Examples of the PCI collision (left) when two cells that are neighbours have the same PCI value and confusion (right) when two neighbouring cells of a cell have the same PCI value^52^.

II. Avoiding PCI collisions and confusions.

reusing the same PCI within a single site or among neighboring cells should be strictly avoided to prevent PCI collisions, where UEs may fail to distinguish between cells, causing synchronization and handover failures. PCI confusion occurs when a cell has two neighbors with the same PCI, leading to ambiguous handover decisions and increased drop rates. Proper PCI assignment directly affects SINR measurements, which influence MCS selection and overall throughput, making careful PCI planning essential for reliable mobility and network performance^52^. The examples of PCI collision, when two cells that are neighbors have the same PCI value, and confusion, when two neighboring cells of a cell have the same PCI value, are presented in Fig. 1.

II. PCI Mod3 and Mod6 Rules.

In 3-sector sites, the same SSS is used for all sectors, while PSS values (0,1,2) ensure unique PCIs, following mod3 planning to reduce intra-site interference. In single-port systems or systems with fewer transmission layers, mod6 planning becomes relevant to avoid interference and maintain efficient signal decoding in mixed MIMO/SISO environments^53,58,55^.

III. PCI Mod30 Rule.

Every 30th PCI shares the same uplink reference signal pattern. Adjacent cells with identical PCI mod30 values may face uplink decoding challenges, potentially increasing block error rates. While rare in commercial networks, this becomes important in dense deployments, so PCI planning must account for mod30 constraints^52,57,58,55^.

IV. PCFICH Collisions.

The Physical Control Format Indicator Channel (PCFICH) location depends on the PCI. Collisions (e.g., every 50th PCI for 20 MHz channels) can lead to decoding failures, impacting UE control channel reception. Intelligent PCI planning is needed to avoid such alignments and ensure system stability^54,55^.

## Optimization algorithms

### Degree of saturation (DSATUR) algorithm

The DSATUR algorithm was originally proposed by Brelaz (1979). It is very similar in behavior to the GREEDY algorithm in that it takes each vertex in turn according to some ordering and then assigns it to the first suitable color class, creating new color classes when necessary. The difference between the two algorithms lies in the way that these vertex orderings are generated. In GREEDY the ordering is decided before any coloring takes place. However, DSATUR algorithm the choice of which vertex to color next is decided heuristically based on the characteristics of the current partial coloring of the graph. This choice is based primarily on the saturation degree of the vertices and it is defined as follows:

#### Definition

Let $\:c\left(v\right)\:=\:NULL$ for any vertex $\:v\:\in\:\:V$ not currently assigned to a colour class. Given such a vertex $\:v$, the saturation degree of $\:v$, denoted by $\:sat\left(v\right)$, is the number of different colours assigned to adjacent vertices. That is,$\:sat\left(v\right)\:=\left|\right\{c\left(u\right)\::\:u\:\in\:\:\varGamma\:\:\left(v\right)\wedge\:c\left(u\right)\:\_=\:NULL\left\}\right|$

### Biased random key genetic algorithms (BRKGA)

**Fig. 2:**
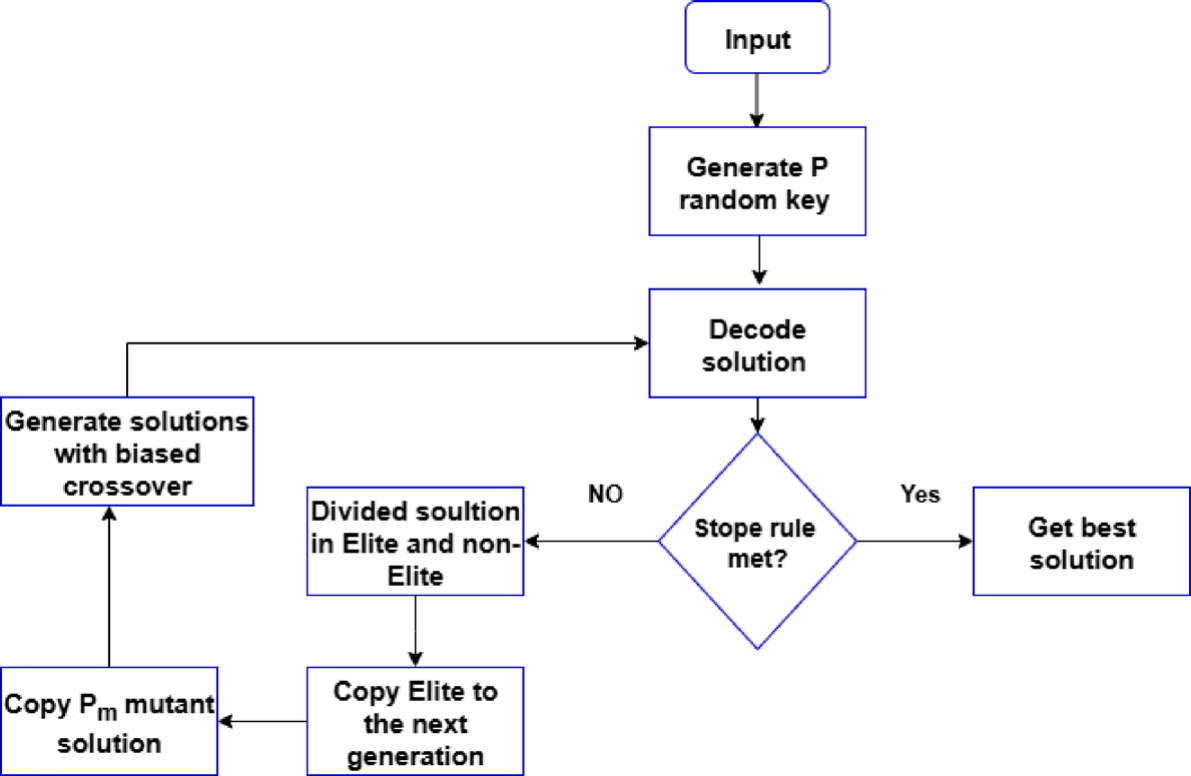
The flow chart of biased random key genetic algorithms.

As seen in Fig. 2, it belongs to the family of search and optimization algorithms known as genetic algorithms, which are motivated by the process of natural selection. The core idea behind GA is to regard the solution to a problem as an individual within a population. A population of possible solutions, each represented by a chromosome or a string of genes, is used by these algorithms. A group of these people makes up the population. The goal of GAs is to mimic nature’s evolutionary process. Through Darwin’s theory of survival of the fittest, the population changes throughout time. While stronger people procreate and spread their traits, weaker individuals are unable to do so and progressively become less prevalent in the community.

There are several generations that the algorithm goes through. Consequently, the algorithm’s output at the end of these generations is the person with the highest fitness value, which frequently indicates the optimal option. The next generation is created by combining individuals from one generation to create children. In most cases, choosing people to form couples based on a probability proportionate to their quality is how the idea of genetic information transmission is put into practice. Stronger individuals are therefore more likely to be selected for reproduction. The mechanism that increases the search and concentrates on passing on the finest qualities of parents to their children is the crossover, which is the process of merging two solutions. After the two parents are chosen, they are somehow united to create children. The genetic information of the offspring is altered by an external mechanism, similar to the mutations seen in nature. Beneficial mutations have the potential to increase the quality of the progeny. Two key elements are included in each search process: diversity and intensity. The primary contribution of the crossover process is intensification, which facilitates the investigation of potential regions within the solution space^22,24^, and^56^. By simulating the process of natural selection, this combination of crossover, mutation, and selection propels population development toward ideal or nearly ideal solutions for the given problem.

### Integer linear programming algorithm (ILP)

**Fig. 3:**
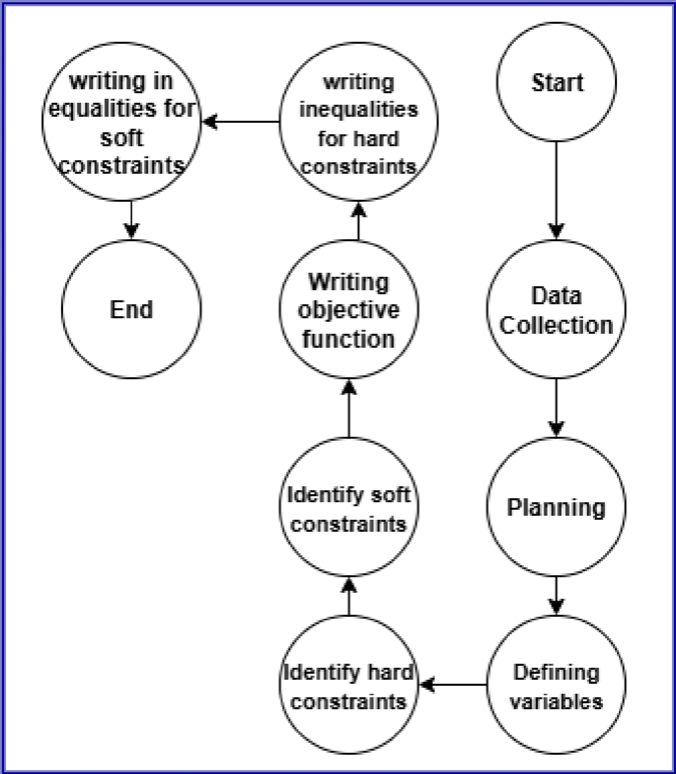
Integer linear programming algorithm.

Figure 3 illustrates that ILP models can be classified based on their variable types into Pure ILP, Mixed-Integer Linear Programming (MILP), and Binary Integer Programming (BIP). In Pure ILP, all decision variables are integers, suitable for problems where partial values are meaningless, such as assigning identifiers or counting discrete units. MILP allows both integer and continuous variables, offering flexibility in systems where binary decisions coexist with real-valued quantities. BIP, a special case of ILP, restricts all variables to binary values (0 or 1) and is ideal for yes/no or selection decisions. In the context of PCI assignment, our problem naturally fits BIP: each variable $\:{x}_{ip}$equals 1 if PCI $\:p$is assigned to cell $\:i$, and 0 otherwise. This binary structure ensures exclusive assignment, satisfying neighbor and top-neighbor constraints while keeping the model straightforward yet effective. Formally, let $\:C$be the set of cells, $\:P=\{\mathrm{0,1},\dots\:,503\}$the valid PCI values, $\:N\subseteq\:C\times\:C$the neighboring cell pairs, and $\:T\subseteq\:C\times\:C$the top neighbors. The PCI assignment problem is then formulated as a BIP to find a feasible assignment that respects all network constraints^57,58^. We formulate the PCI assignment problem as an Integer Linear Programming (ILP) model with the objective of finding a feasible assignment that satisfies all neighbor constraints.

**I. Decision Variables**.

1. $\:{x}_{i,p}\in\:\left\{\mathrm{0,1}\right\}$– binary variable indicating whether PCI $\:p$is assigned to cell $\:i$.
2. $\:{m}_{i}\in\:\{\mathrm{0,1},2\}$– Primary Synchronization Signal (PSS) for cell $\:i$.
3. $\:{s}_{i}\in\:\left[\mathrm{0,167}\right]$– Secondary Synchronization Signal (SSS) for cell $\:i$.

**II. Objective Function**

$$\:\mathrm{Minimize:\:}0\mathrm{(we\:aim\:to\:find\:a\:feasible\:solution\:satisfying\:all\:constraints)}$$.

**III. Constraints**.

One PCI per cell

4 $$\:\sum\:_{p=0}^{503}{x}_{i,p}=1,\forall\:i\in\:\mathrm{Cells}$$

Link PCI to PSS and SSS

5 $$\:{m}_{i}=\sum\:_{p=0}^{503}\left(p{\hspace{0.05em}}mod{\hspace{0.05em}}3\right)\cdot\:{x}_{i,p},{s}_{i}=\sum\:_{p=0}^{503}\lfloor\:p/3\rfloor\:\cdot\:{x}_{i,p},\forall\:i$$

Regular neighbor constraint

6 $$\:{x}_{i,p}+{x}_{j,p}\le\:1,\forall\:(i,j)\in\:\mathrm{neighbors},\forall\:p$$

4. Top neighbors’ constraint.

- Different PCI:

7 $$\:{x}_{i,p}+{x}_{j,p}\le\:1,\forall\:(i,j)\in\:\mathrm{top\_neighbors},\forall\:p$$

- Different PSS:

8 $$\:1\le\:{m}_{i}-{m}_{j}+3{\delta\:}_{ij}\le\:2,{\delta\:}_{ij}\in\:\left\{\mathrm{0,1}\right\}$$

(Optional/Removed) Different SSS for all cells Constraint commented out due to infeasibility:

9 $$\:{s}_{i}-{s}_{j}+M{\delta\:}_{ij}^{SSS}\ge\:1,{s}_{i}-{s}_{j}-M(1-{\delta\:}_{ij}^{SSS})\le\:-1$$

This prevents the reuse of the same PCI between adjacent cells. Then define auxiliary integer variables: Let $\:{m}_{i}\in\:\mathrm{0,1},2$ be the PSS of the PCI assigned to cell $\:i$ and $\:{s}_{i}\in\:0,\cdots\:,167$ be the SSS of the PCI assigned to cell $\:i$ and it can computed as:

10 $$\:{s_i} = {\sum \: _{p \in \:P}}{x_{ip}} \cdot \:\left( {P/3} \right)\;$$

These expressions convert PCI assignments into their respective components. PSS difference between top neighbors. To enforce $\:{m}_{i}\ne\:{m}_{j}$ for all $\:\left(i,j\right)\:\epsilon\:T$ we use a binary auxiliary variable $\:{\delta\:}_{ij}\in\:\mathrm{0,1}$ and write:

11 $$\:{m}_{i}-{m}_{j}+3{\delta\:}_{\mathrm{i}j}\le\:\:2\:\mathrm{a}\mathrm{n}\mathrm{d}\:{m}_{j}-{m}_{i}+3\left(1-{\delta\:}_{ij}\right)\le\:2$$

Ensures distinct SSS values: In high-interference environments, we may require:

12 $$\:{s}_{i}\ne\:{s}_{j}\:\:\:\:\forall\:\left(\mathrm{i},j\right)\in\:c$$

which is linearized similarly using delta variables if necessary. This complete formulation represents a Binary Integer Program (BIP), as all decision variables $\:{x}_{ip}$ are binary and constraints are linear. According to standard ILP modeling principles, such formulations are solvable by branch-and-bound algorithms and linear relaxation techniques^59^.

### Complexity analysis

The computational complexity of the evaluated PCI allocation methods is discussed in a theoretical and comparative sense, rather than using absolute runtime values, as execution time is highly dependent on hardware and solver configurations. ILP exhibits exponential complexity with respect to the number of cells, which explains its inability to scale to large networks despite guaranteeing an optimal solution. For this reason, ILP is used solely as an optimal benchmark in this study. Furthermore, DSATUR has polynomial complexity and low memory requirements, making it computationally efficient and suitable for small to medium network sizes, albeit without global optimality guarantees. Additionally, BRKGA introduces higher computational overhead due to its population-based nature, but it scales significantly better than ILP and consistently achieves near-optimal solutions in dense scenarios. These observations highlight an inherent complexity–performance trade-off, which is the focus of this work. The results confirm that algorithm selection should be guided by network scale and operational constraints rather than theoretical optimality alone. Table 1 gives comparison between them.

**Table 1:** Comparison between different optimization algorithms according to complexity.

Method	Time Complexity	Memory Usage	Expected PSS Conflicts	Advantages	Disadvantages
ILP	Exponential in number of cells $\:\left(O\right({3}^{N}\left)\right)$	High (stores all constraints)	optimal	Guarantees optimal solution, minimal conflicts	Very slow for large networks, impractical for *N* > 100
DSATUR	$\:\left(O\right({N}^{2}\times\:\:MA{X}_{PCI}\left)\right)$	Low to medium	Low (heuristic)	Fast, easy to implement, good for medium networks	May not find global optimum, some conflicts possible
BRKGA_MP	Depends on generations and population size (roughly $\:\left(O\right(G\times\:\:P\times\:\:{N}^{2}\left)\right))$	Medium to high	Very low (near optimal)	Can escape local minima, flexible for large networks	Needs parameter tuning, slower than DSATUR, stochastic results

### Simulation results

Before downloading NS3 source code and running it, first some dependencies must be installed. The dependencies are listed in Table 2:

**Table 2:** Required packages to run NS3.

Dependencies	Package/Version
C + + Compiler	clang++ or g++ (g + + version 9 or greater)
Python	python3 version > = 3.8
CMake	cmake version > = 3.13
Build System	make, ninja, xcodebuild (XCode)
Git	Any recent version (to access ns-3 from GitLab.com)
tar	Any recent version (to unpack an ns-3 release)
bunzip2	Any recent version (to uncompress an ns-3 release)

This section presents the results obtained from NS3 simulation running the script. The script would first run without any optimized PCI allocation solution. The network graph is extracted from this unoptimized run from the ANR and making relevance to handovers between cells. The network graph is used to obtain an optimum PCI allocation using one of the three optimization algorithms described above. Finally, the simulation script is run again, and the two runs are compared according to the SINR trace provided by each one of them. The primary Key Performance Indicator (KPI) used to evaluate the efficacy of the optimized PCI allocation solutions against the unoptimized baseline is the Signal-to-Interference-plus-Noise Ratio (SINR). An enhancement in network SINR serves as a direct measure of interference mitigation and translates to an improved Quality of Service (QoS) for the end-user. Our analysis specifically focuses on the relative frequency of SINR measurements falling into the “Excellent” category (> = 20 dB). Our results show that in smaller networks of 6 to 18 eNodeBs, optimization algorithms can achieve substantial gains, with DSATUR delivering a peak improvement of over 25% points. However, this enhancement is highly dependent on network scale, with the average improvement across all scenarios and algorithms being closer to 5% points.

### Experimental setup and methodology

I. Simulation Environment.

We used NS3 as the simulation environment for our work. NS3 provides simulation modules for both LTE and NR. LTE modules were used because of their available extensive documentation. The Programming languages used are C + + for NS3 simulation and DSATUR optimization algorithm. Python was used for the Three remaining optimization algorithms, namely BRKGA and ILP algorithms. The installation of NS3 was described in Chap. 8 along with the needed libraries. The libraries needed for the optimization algorithms are NumPy, matplotlib, collections, network, pandas and pulp. The simulation was done on Ubuntu Linux operating system installed on a virtual machine using Oracle virtual box. 6 cores were allocated to this virtual machine from an Intel(R) Core (TM) i5-11400 H @ 2.70 GHz CPU with RAM memory of 5GB. The allocated storage was 50GB SSD. Simulations were also run on a device with 11th Gen Intel(R) Core (TM) i7-11800 H @ 2.30 GHz 2.30 GHz CPU where 7 cores were used for the virtual environment. 11GB of RAM was also allocated to the virtual machine. However, the simulation explicitly configures UE mobility and system bandwidth in NS-3 as follows:

1. UE Speed Configuration.
2. User equipment (UE) velocities are randomly distributed between 0 m/s and 5 m/s, representing realistic low-to-moderate mobility scenarios. In NS-3, this is implemented as:

velocityRandom-> SetAttribute(“Min”, DoubleValue(0));//Minimum velocity (m/s).

velocityRandom-> SetAttribute(“Max”, DoubleValue(5.0));//Maximum velocity (m/s).

3. System Bandwidth Configuration.
4. The LTE system uses 100 resource blocks (RBs), corresponding to 20 MHz bandwidth, for both uplink and downlink. In NS-3, this is configured as:

lteHelper-> SetEnbDeviceAttribute(“DlBandwidth”, UintegerValue(100));//Downlink RBs.

lteHelper-> SetEnbDeviceAttribute(“UlBandwidth”, UintegerValue(100));//Uplink RBs.

These configurations ensure that the simulations capture realistic mobility and radio resource conditions, providing a reliable foundation for evaluating PCI allocation algorithms under practical network scenarios.

II. Used algorithms

3 Different algorithms were used in this work, namely DSATUR, biased random key genetic BRKGA and ILP. The latter was lately modified to operate with clustering to make the algorithm able to solve larger networks. All of this will be discussed in the following sections.

III. Baseline Scenario: Iterative PCI Allocation

The scenario starts with the configured number of eNodeBs and number of UEs. The number of UEs is fixed at 10 UE per eNodeB. The simulation run time is 30 seconds. High simulation durations would be infeasible and would require higher computational resources. The number of bearers that could be assigned to a single UE is 2 bearers. The Basic Penalty factor introduced by us and the PCI penalty Scaling factor are set to 0 and 1 respectively. The base Noise Figure (NF) is set to 16dB. The data rate of the point-to-point channel initiated between the EPC and the remote host is 10Gbps which is suitable for this scenario. The data rate of a single eNodeB can provide up to 100Mbps in the LTE standards. The Maximum Transmission Unit (MTU) is set to 1000 byte. IP allocations and similar attributes are not relevant and can be set according to any scheme. The uplink and downlink carriers are set to the maximum allowed by the standards which is 20MHz(100RBs). We set it to this value as we did not want to face any data rates bottle nick because this is not our focus. Each of the UEs initiates its service according to a random variable between 0.05 seconds and 10 seconds. Lower values would cause MAC scheduler saturation as it would receive a lot of initial access messages. The Evolved Packet System (EPS) bearer setup for the UEs is GBR_CONV_VOICE which corresponds to QOS Class Identifier (QCI) of 1. Finally, the RSRQ threshold value for Automatic Neighbour Relations (ANR) neighbour addition to the Network Relation Table (NRT) is set to 20. RSRQ measured values in dB are usually mapped to quantized values from 0 to 34, where each step represents a range of 0.5 dB. So, 20 represents an RSRQ value between -10dB and -9.5dB. this is an Excellent RSRQ value. The unoptimized run of the script is done using iterative PCI allocations. This means that the first registered eNodeB takes a PCI value of 1, the second takes a PCI of 2 and so on. NS3 does not allow PCI values of 0 or two eNodeBs to have the same PCI. This limitation exists due to the NS3 coupling between PCI and Cell-ID. Cell-ID is a unique global identifier that identifies the cell on the network, so no two eNodeBs can be assigned the same Cell-ID. Changing this would require decoupling the two attributes in NS3 which is very difficult due to the convoluted nature of the source code. Thus, we chose to work with this limitation and study the effect of MOD3 PCI only. The mobility model installed on eNodeBs is a static mobility model obviously. The mobility model installed on the UEs is random direction 2D mobility model. In this mobility model the UEs move with a random velocity at a random direction until it hits a rectangular boundary where it stops and starts moving again with a random velocity and a random direction. The eNodeBs is placed in a rectangular grid with 1Km interspacing. The rectangular boundaries of the UEs are set with width and height of 3Km. This value ensures that UE would traverse from one eNodeB coverage to another one to initiate handovers in the simulation 

IV. Network Graph Generation

Each eNodeB in the simulation represents a graph node in the network graph. An edge relation between two nodes is extended if two nodes are entries of the neighbors relations table (NRT). Entries of the NRT table are generated during the run of the unoptimized script according to the ANR algorithm. This represents a real network scenario running for the same time to generate the NRT according to UEs mobility and then generate the optimized PCI Allocation. Two Nodes(eNodeBs) are considered top neighbours if a handover happens between them. In future research the top neighbor relation between nodes might be weighted according to the number of handovers that occurred. The ANR algorithm used is the one employed by NS3. If a measured RSRQ from a certain eNodeB exceeds the threshold given above, this eNodeB is added to the neighbours of the eNodeB that the UE is connected to. Future research can be done on the optimal value on the ANR threshold.

V. Evaluation Workflow.

- The baseline Simulation script is run as the first step with a specified number of eNodeBs (16 for example).
- The UE Traces is generated (UEs Trajectories and the corresponding SINR at each point).
- The NRT table and Top neighbours table is extracted according to the algorithms specified above.
- One of the optimization algorithms is executed using the NRT table and the Top neighbor’s table. (ILP for example).
- The optimization algorithm generates an optimum PCI allocation for the network generated.
- The same script is executed again, but this time with the optimized PCI allocation.
- All the previous steps are repeated for the other two optimization algorithms.

**Algorithm 1:**
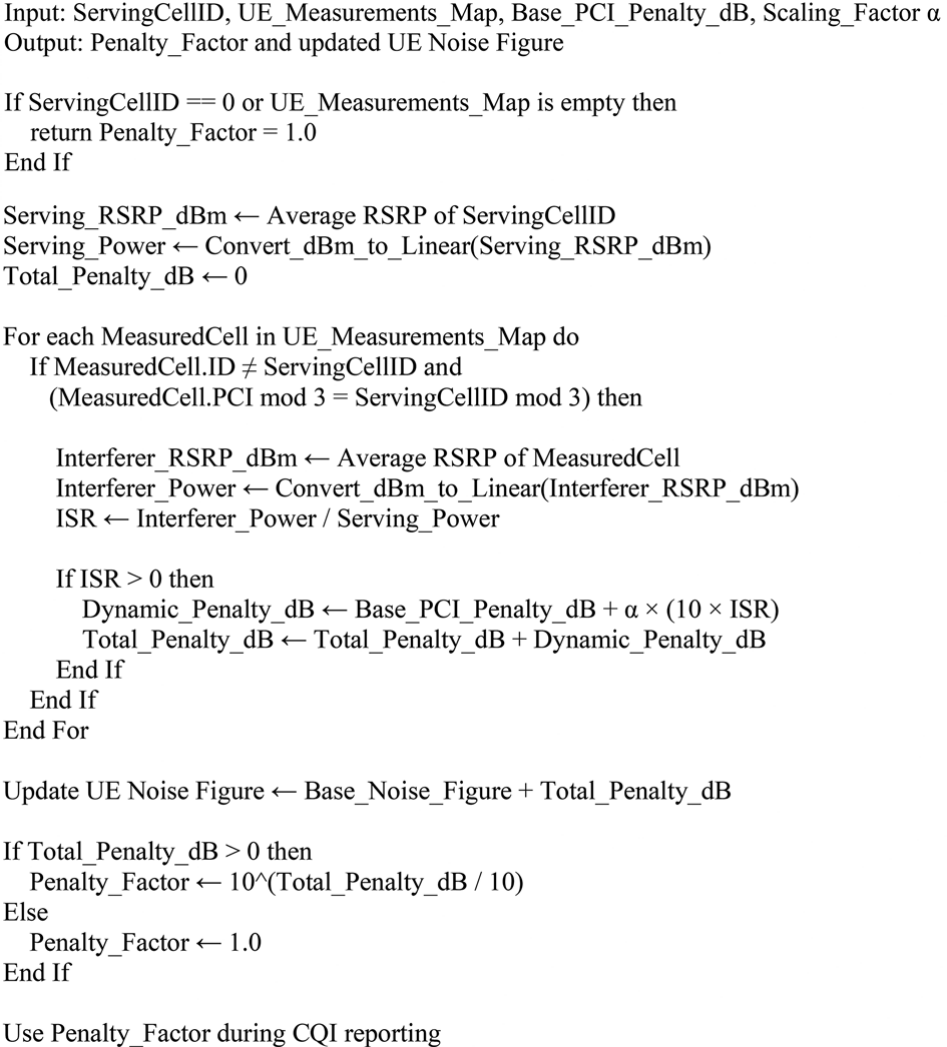

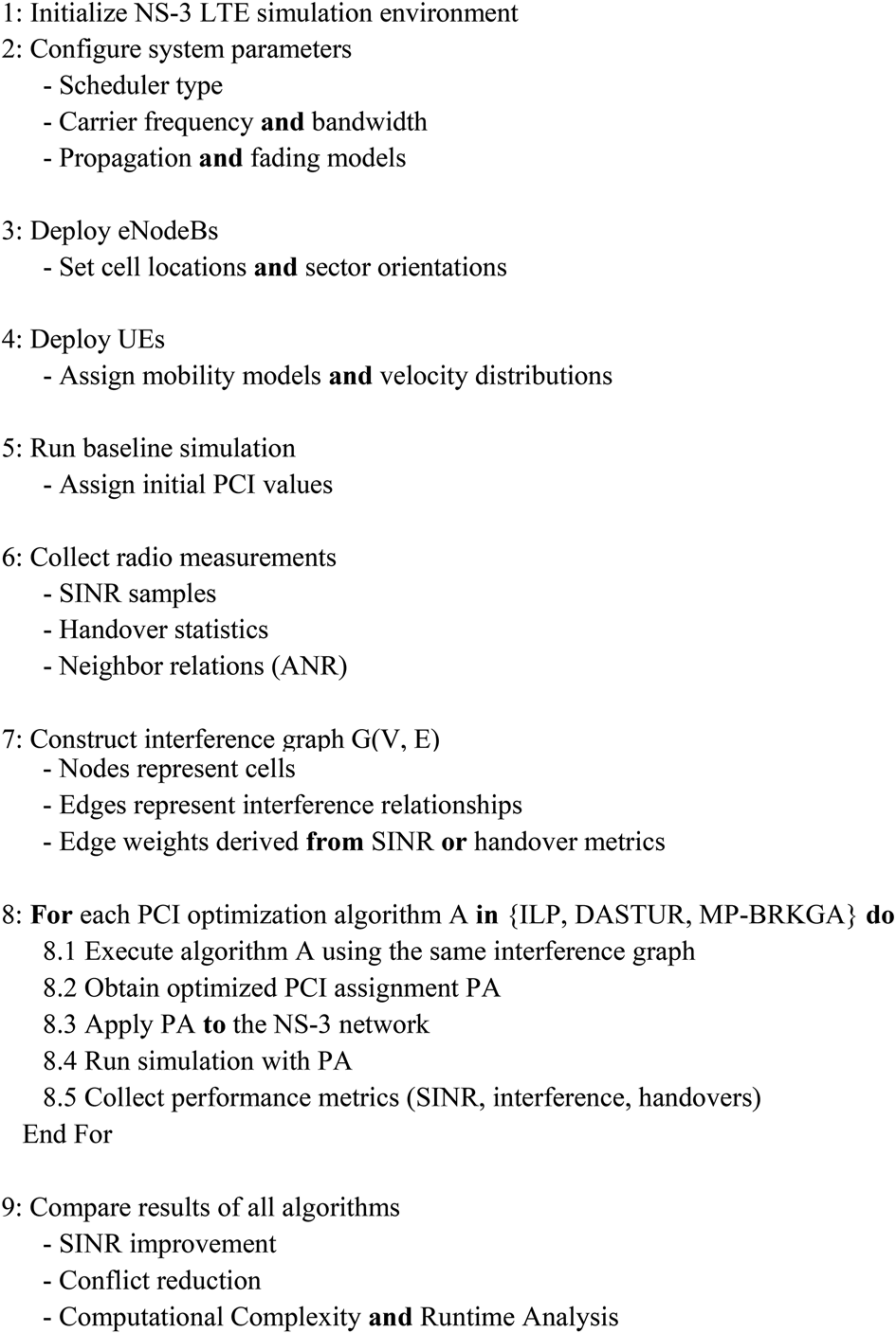
The pseudo code below shows the evaluation workflow of our experimentation and penalty: Penalty pseudo code.

**Pseudo-Code 1:**
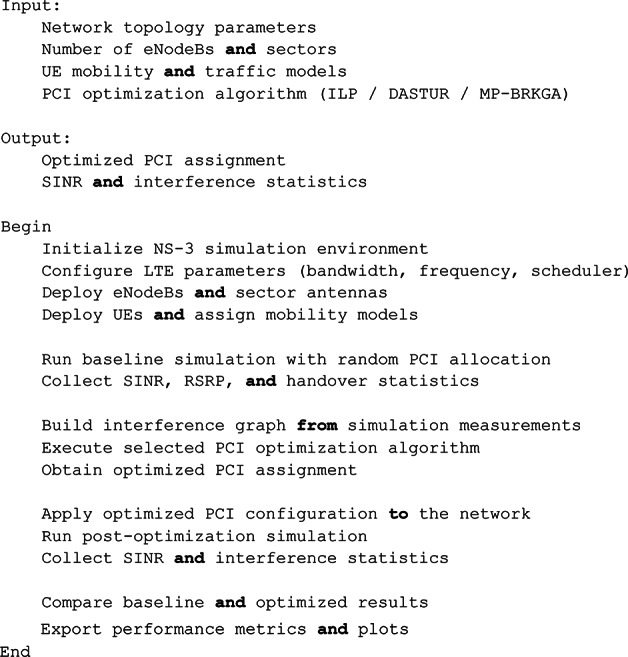
Overall Simulation and PCI Optimization Flow. Main Simulation Workflow.

**Pseudo-Code 2:**
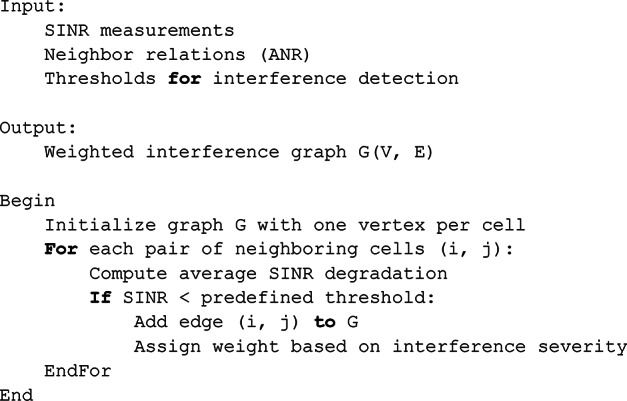
Interference Graph Construction.

**Pseudo-Code 3:**
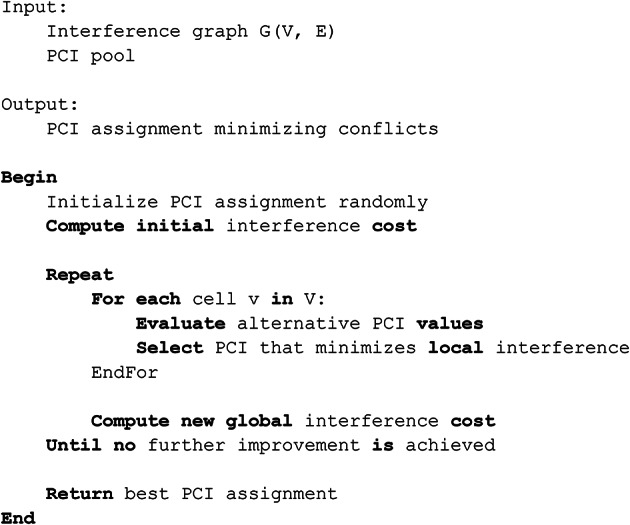
DASTUR Algorithm (Iterative Heuristic).

**Pseudo-Code 4:**
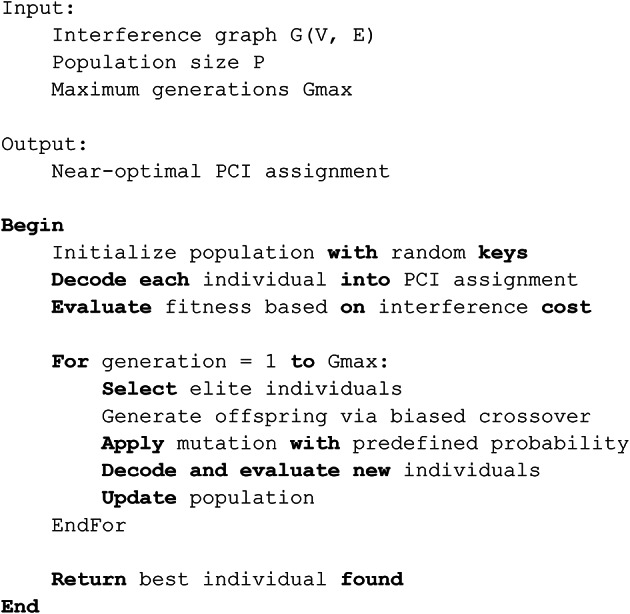
MP-BRKGA Algorithm.

**Pseudo-Code 4:**
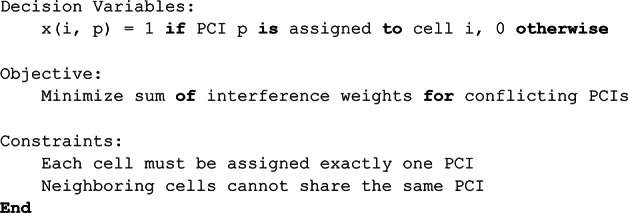
MP-BRKGA Algorithm.

### Initial performance analysis

This section shows the values of SINR before any optimization is done. It is crucial to see the effects of bad PCI planning and how it affects the quality of service given to users.

### Initial SINR distribution

**Fig. 4:**
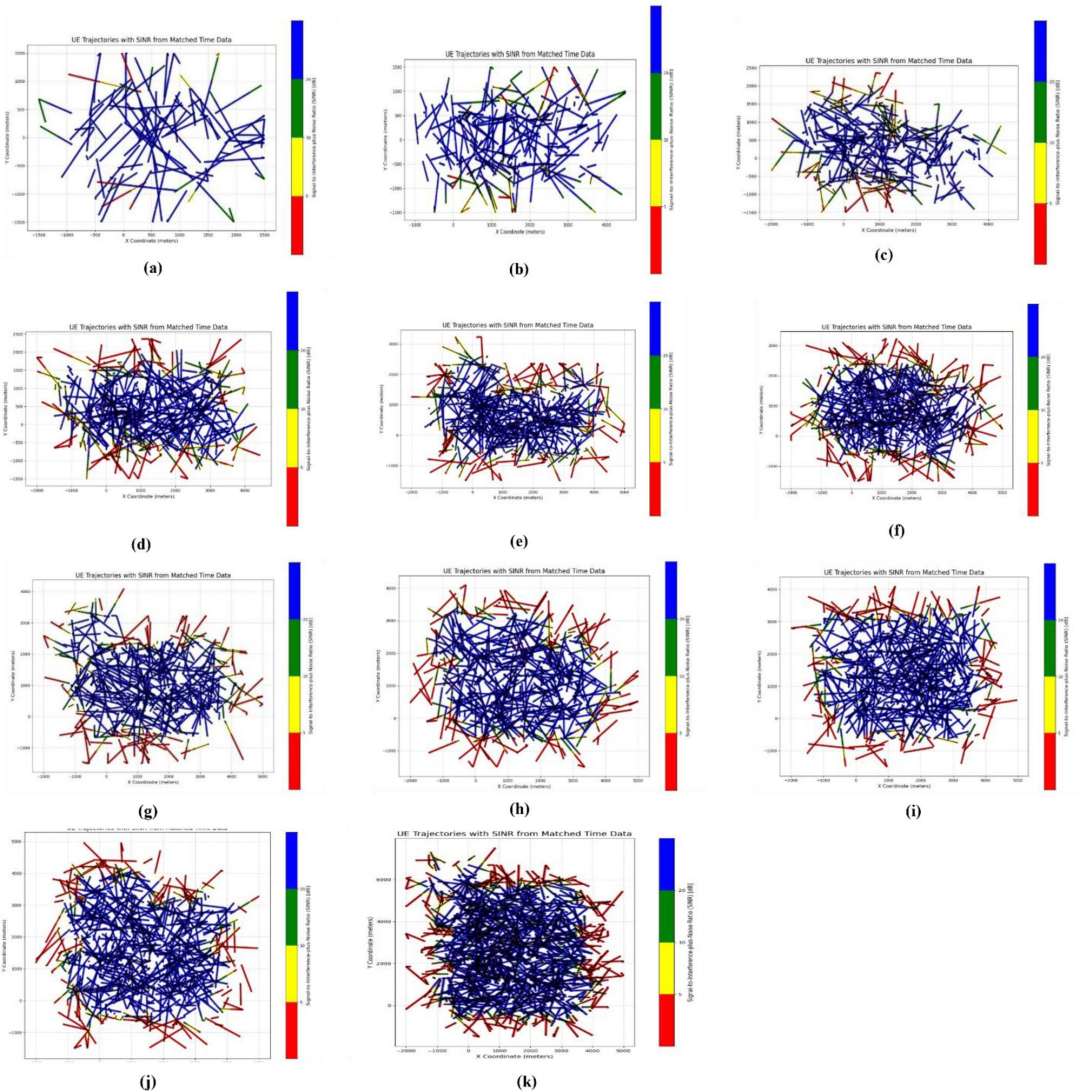
Random movement of UE trajectories at different number of eNodeBs for initial SNR distribution ((**a**) 6eNodeBs, (**b**) 12eNodeBs, (**c**) 18eNodeBs, (**d**) 24eNodeBs, (**e**) 30eNodeBs, (**f**) 36eNodeBs, (**g**) 42eNodeBs, (**h**) 48eNodeBs, (**i**) 54eNodeBs, (**j**) 60eNodeBs, (**k**) 96eNodeBs).

**Fig. 5:**
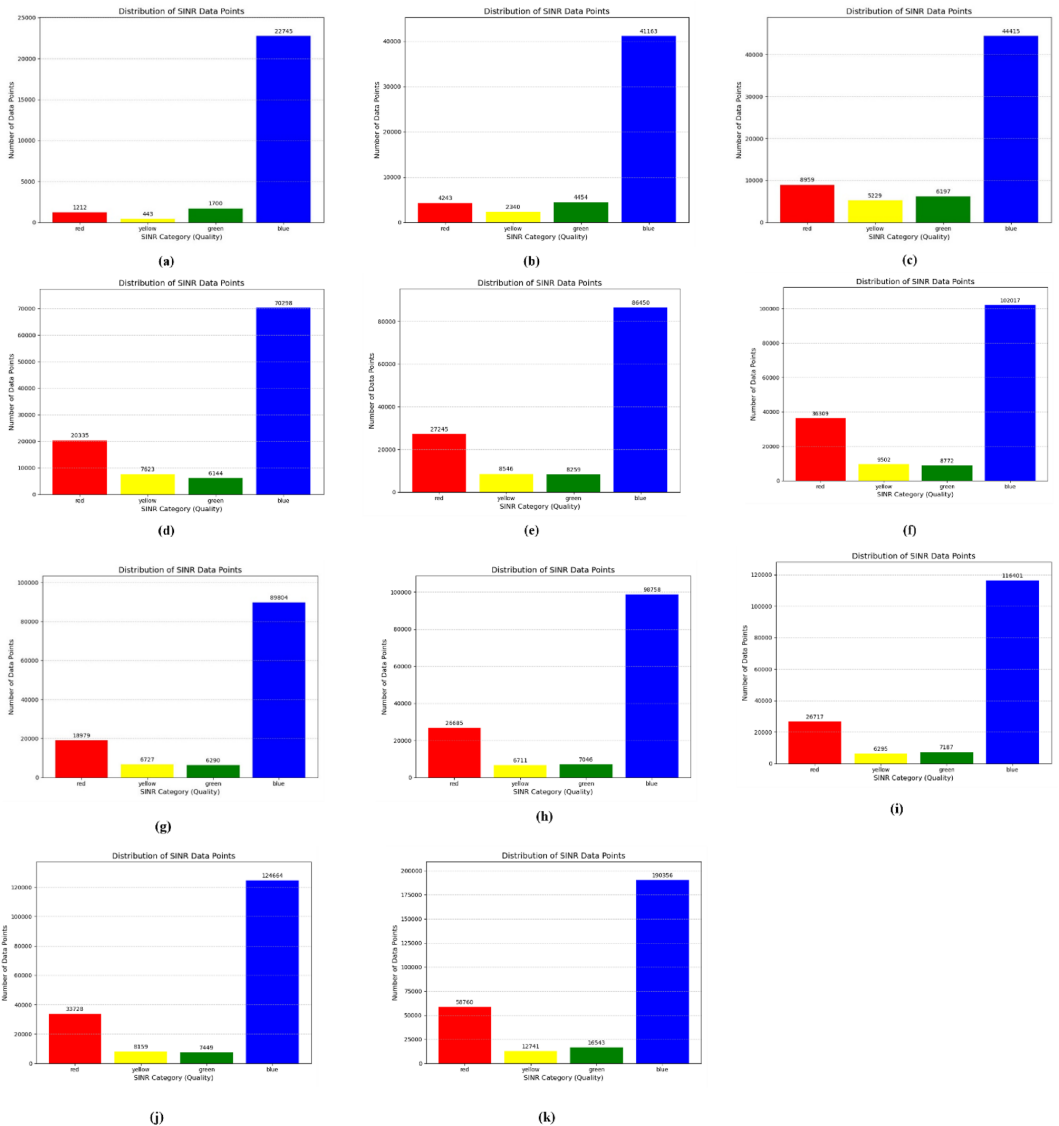
Number of SINR points at different level *(*(**a**) 6eNodeBs, (**b**) 12eNodeBs, (**c**) 18eNodeBs, (**d**) 24eNodeBs, (**e**) 30eNodeBs, (**f**) 36eNodeBs, (**g**) 42eNodeBs, (**h**) 48eNodeBs, (**i**) 54eNodeBs, (**j**) 60eNodeBs, (**k**) 96eNodeBs).

The initial distribution of SINR is shown in the following figures, with different number of eNodeBs. The first figure shows the trajectories of user equipment along with the corresponding value of SINR at each point. The color coding is done to separate SINR according to Table 3. The X axis of the figure represents the distance of the UE from the origin point along the X axis, while the Y axis represents the distance of the UE from the origin point along the Y axis. Figure 4 shows these trajectories. The plots above show a distinctive pattern of SINR. As it is clear, UE at the edges of the Network has very low SINR. This is an understandable result as these UEs are practically outside the coverage of the network. This happened due to the rectangular boundaries on the UEs moving which extend outside the coverage of the network to allow for Extensive UE mobility inside the network. One can also see parts of the trajectories in the middle of the network with bad SINR due to interference. The portion of bad SINR increases as the number of eNodeBs increases. This is due to the introduction of mod3 interfering with eNodeBs. Furthermore, Figs. 4 and 5 show the frequency distribution of SINR with different numbers of eNodeBs. In addition, Tables 4 and 5 show the percentage of blue, green, yellow and red SINR points.

**Table 3:** SINR ranges.

SINR > 20dB	Excellent
10dB > SINR > 20dB	Good
5dB < SINR < 10dB	Fair
SINR < 5dB	Bad

**Table 4:** Number of data points at different SINR points.

SINR Category	SINR Range (dB)	Number of Data Points					
		36eNodeB	42eNodeB	48eNodeB	54eNodeB	60eNodeB	96eNodeB
Red	< 5	36,309	18,979	26,685	26,717	33,728	58,760
Yellow	5 to 10	9502	6727	6711	6295	8159	12,741
Green	10 to 20	8772	6290	7046	7187	7449	16,543
Blue	>= 20	102,017	89,804	98,758	116,401	124,664	190,356

**Table 5:** Relative frequency at different SINR points.

SINR Category	SINR Range (dB)	Relative Frequency (%)					
		36eNodeB	42eNodeB	48eNodeB	54eNodeB	60eNodeB	96eNodeB
Red	< 5	23.19	15.58	19.17	17.06	19.38	21.11
Yellow	5 to 10	6.07	5.52	4.82	4.02	4.69	4.58
Green	10 to 20	5.6	5.16	5.06	4.59	4.28	5.94
Blue	>= 20	65.14	73.73	70.95	74.33	71.65	68.38

As it is clear the percentage of blue decreases as the number of eNodeBs increases due to the two reasons discussed above (UEs at the edge of the network and emergent MOD3 PCI eNodeBs).

### Performance of PCI optimization algorithms

Having established the performance benchmarks and limitations of the initial iterative PCI allocation in the preceding section, this section now evaluates the effectiveness of the three proposed optimization algorithms. The primary objective is to quantify the improvement in network performance resulting from intelligent PCI planning based on the network handover graph. To provide a clear and systematic analysis, each algorithm is assessed in a dedicated subsection. The core metric for this evaluation is the UE SINR. For each algorithm, the resulting SINR distribution will be presented and directly compared against the baseline scenario to highlight the magnitude of the enhancement, particularly for users at the cell edge. This individual assessment will form the foundation for the comprehensive comparative analysis in the following sections.

### Degree of saturation (DSATUR) results

**Fig. 6:**
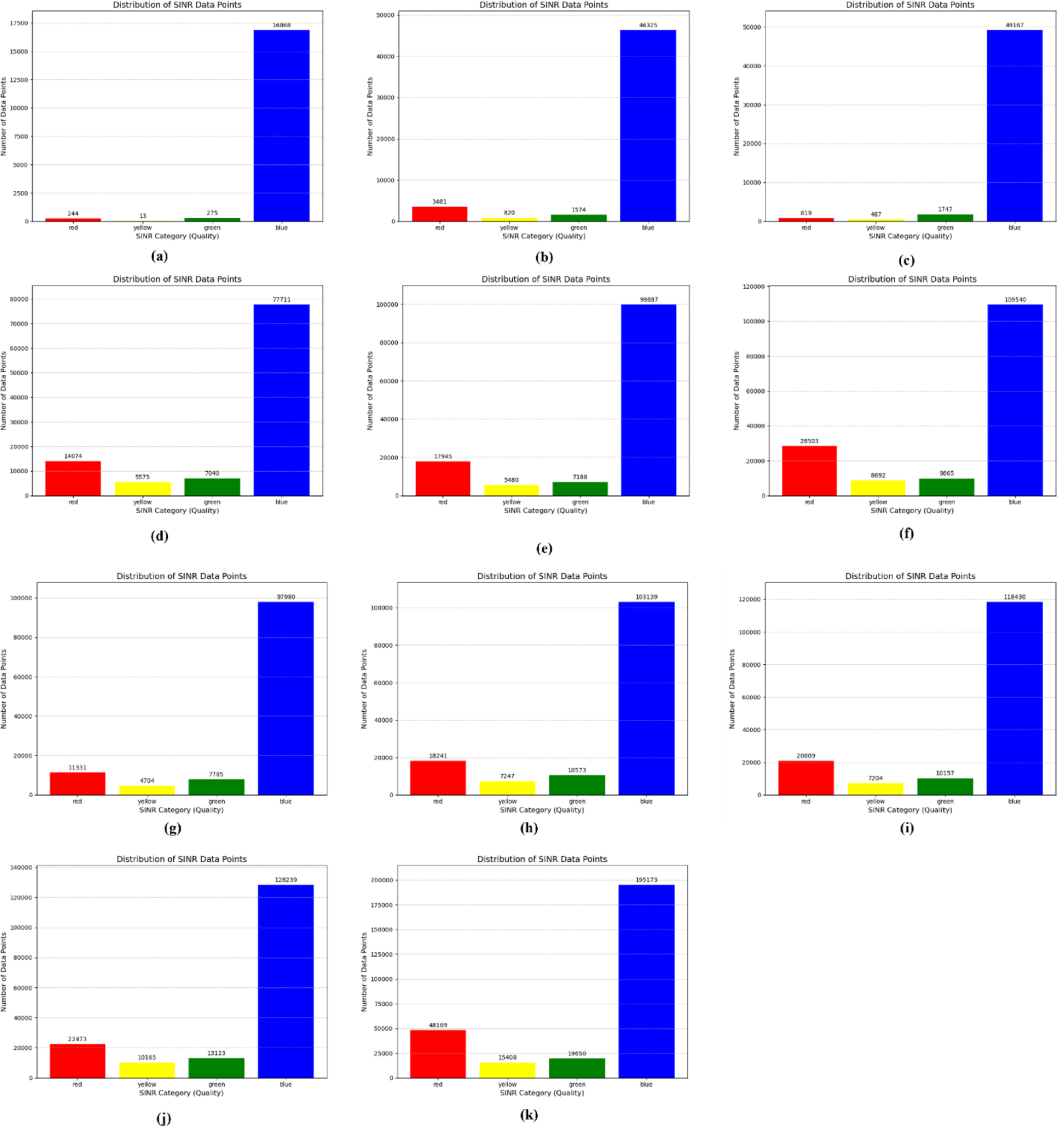
Random movement of UE trajectories at different number of eNodeBs using DUSTAR algorithm ((**a**) 6eNodeBs, (**b**) 12eNodeBs, (**c**) 18eNodeBs, (**d**) 24eNodeBs, (**e**) 30eNodeBs, (**f**) 36eNodeBs, (**g**) 42eNodeBs, (**h**) 48eNodeBs, (**i**) 54eNodeBs, (**j**) 60eNodeBs, (**k**) 96eNodeBs).

**Fig. 7:**
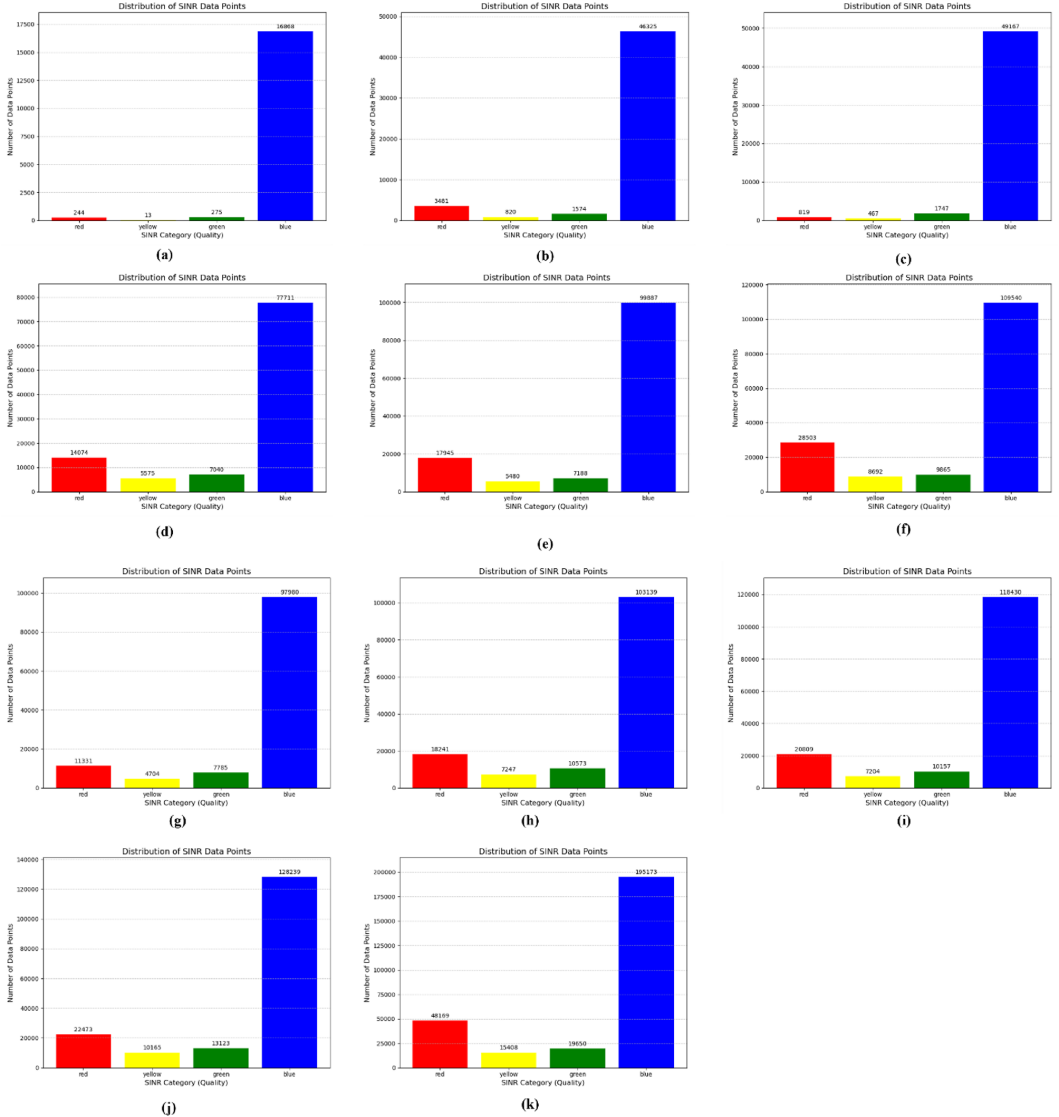
Number of SINR points at different level for DUSTAR (**a**) 6eNodeBs, (**b**) 12eNodeBs, (**c**) 18eNodeBs, (**d**) 24eNodeBs, (**e**) 30eNodeBs, (**f**) 36eNodeBs, (**g**) 42eNodeBs, (**h**) 48eNodeBs, (**i**) 54eNodeBs, (**j**) 60eNodeBs, (**k**) 96eNodeBs).

 Figure 6 illustrates the UE trajectories and corresponding SINR points for the optimized scenario. An improvement in SINR is observed in the central region of the network due to reduced PCI MOD3 interference, as well as for edge users. Figure 7 presents the SINR distribution across the four color-coded classes, followed by Tables 6 and 7, which report the corresponding percentages and compare them with the baseline scenario shown in Table 5. Overall, the results indicate a clear SINR improvement across most scenarios, although the performance gain decreases as the network size increases. For larger networks, BRKGA demonstrates superior performance. In addition, Table 8 summarizes the relative frequency at different SINR points for DASTUR.

**Table 6:** Relative frequency at different SINR points.

SINR Category	SINR Range (dB)	Relative Frequency (%)				
		6eNodeB	12eNodeB	18eNodeB	24eNodeB	30eNodeB
Red	< 5	4.64	8.13	13.83	19.48	20.88
Yellow	5 to 10	1.7	4.48	8.07	7.3	6.55
Green	10 to 20	6.51	8.53	9.56	5.89	6.33
Blue	>= 20	87.15	78.86	68.54	67.34	66.25

**Table 7:** Number of data points at different SINR points for DASTUR.

SINR Category	SINR Range (dB)	Number of Data Points										
		6eNodeB	12eNodeB	18eNodeB	24eNodeB	30eNodeB	36eNodeB	42eNodeB	48eNodeB	54eNodeB	60eNodeB	96eNodeB
Red	< 5	244	3481	819	14,074	17,945	28,503	11,331	18,241	20,809	22,473	48,169
Yellow	5 to 10	13	820	467	5575	5480	8692	4704	7247	7204	10,165	15,408
Green	10 to 20	275	1574	1747	7040	7188	9865	7785	10,573	10,157	13,123	19,650
Blue	>= 20	16,868	46,325	49,167	77,711	99,887	109,540	97,980	103,139	118,430	128,239	195,173

**Table 8:** Relative frequency at different SINR points for DASTUR.

SINR Category	SINR Range (dB)	Relative Frequency (%) DSATUR										
		6eNodeB	12eNodeB	18eNodeB	24eNodeB	30eNodeB	36eNodeB	42eNodeB	48eNodeB	54eNodeB	60eNodeB	96eNodeB
Red	< 5	1.4023	6.67	1.56	13.48	13.75	18.2	9.302	13.1	13.29	12.92	17.3
Yellow	5:10	0.0747	1.57	0.89	5.34	4.2	5.55	3.86	5.21	4.6	5.84	5.53
Green	10:20	1.58	3.02	3.3467	6.74	5.51	6.3	6.39	7.6	6.49	7.54	7.06
Blue	>= 20	96.9425	88.75	94.1896	74.44	76.54	69.95	80.44	74.09	75.63	73.7	70.11

**Fig. 8:**
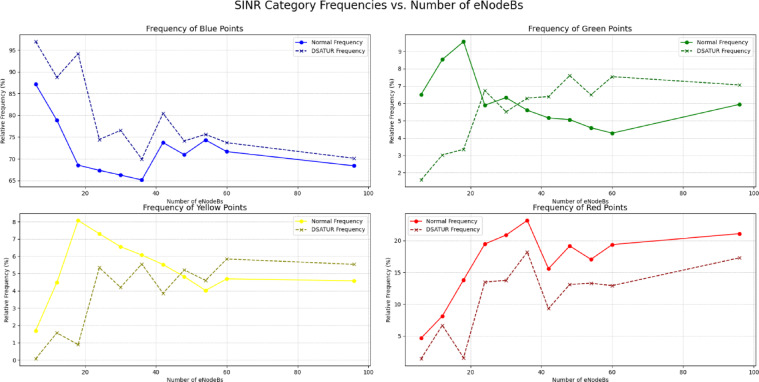
SINR comparison between DSATUR and Normal iteration).

**Fig. 9:**
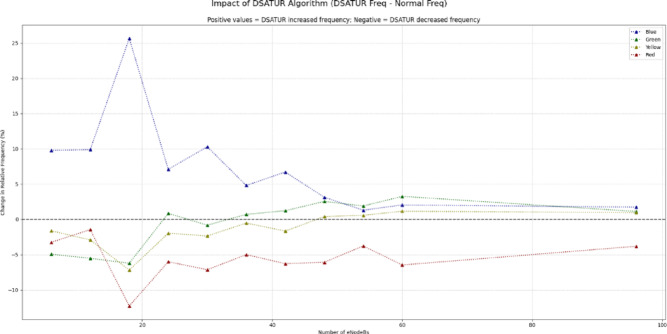
Difference between DSATUR and Normal run (SINR).

Although some runs do not improve the SINR very much, especially at large node numbers, the solution provides a clear decrease in red SINR class. It also provides an apparent increase in all the blue, green and yellow SINR classes. The enhancement in system performance by increasing the SINR perceived by the mobile can be increased also as we have found by changing the value of the ANR threshold. As this value clearly controls the number of neighbours seen by the eNodeBs in the network, decreasing it would allow the optimization algorithms to provide better SINR improvement. At the same time, decreasing the threshold would provide more complex graph structures which would result in longer computational time for the algorithms to provide an optimal solution. So, the trade-off between the quality of the solution and the run time should be considered. However, this is left for future development. Figure 8 presents SINR comparison while Fig. 9. shows a complete overview of network enhancement. DSATUR algorithm provides enhancement in the value of SINR along with all the number of nodes tested. It approaches the value of base simulation SINR. This fact makes the use of BRKGA more prominent in large networks sizes. Our deployment program will consider this fact along with the run time of each algorithm.

### BRKGA results

**Fig. 10:**
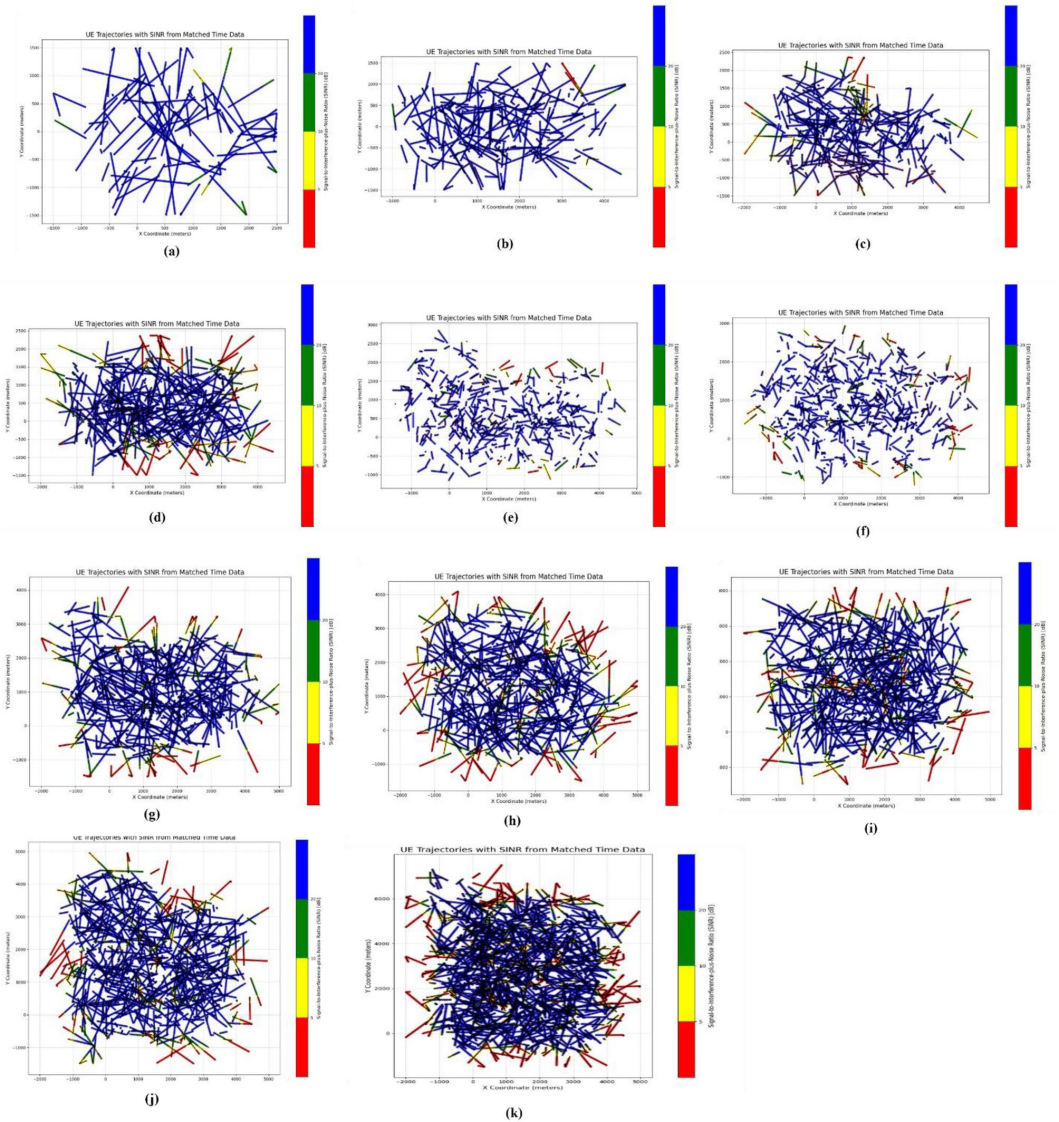
Random movement of UE trajectories at different number of eNodeBs using BRKGA algorithm ((**a**) 6eNodeBs, (**b**) 12eNodeBs, (**c**) 18eNodeBs, (**d**) 24eNodeBs, (**e**) 30eNodeBs, (**f**) 36eNodeBs, (**g**) 42eNodeBs, (**h**) 48eNodeBs, (**i**) 54eNodeBs, (**j**) 60eNodeBs, (**k**) 96eNodeBs).

**Fig. 11:**
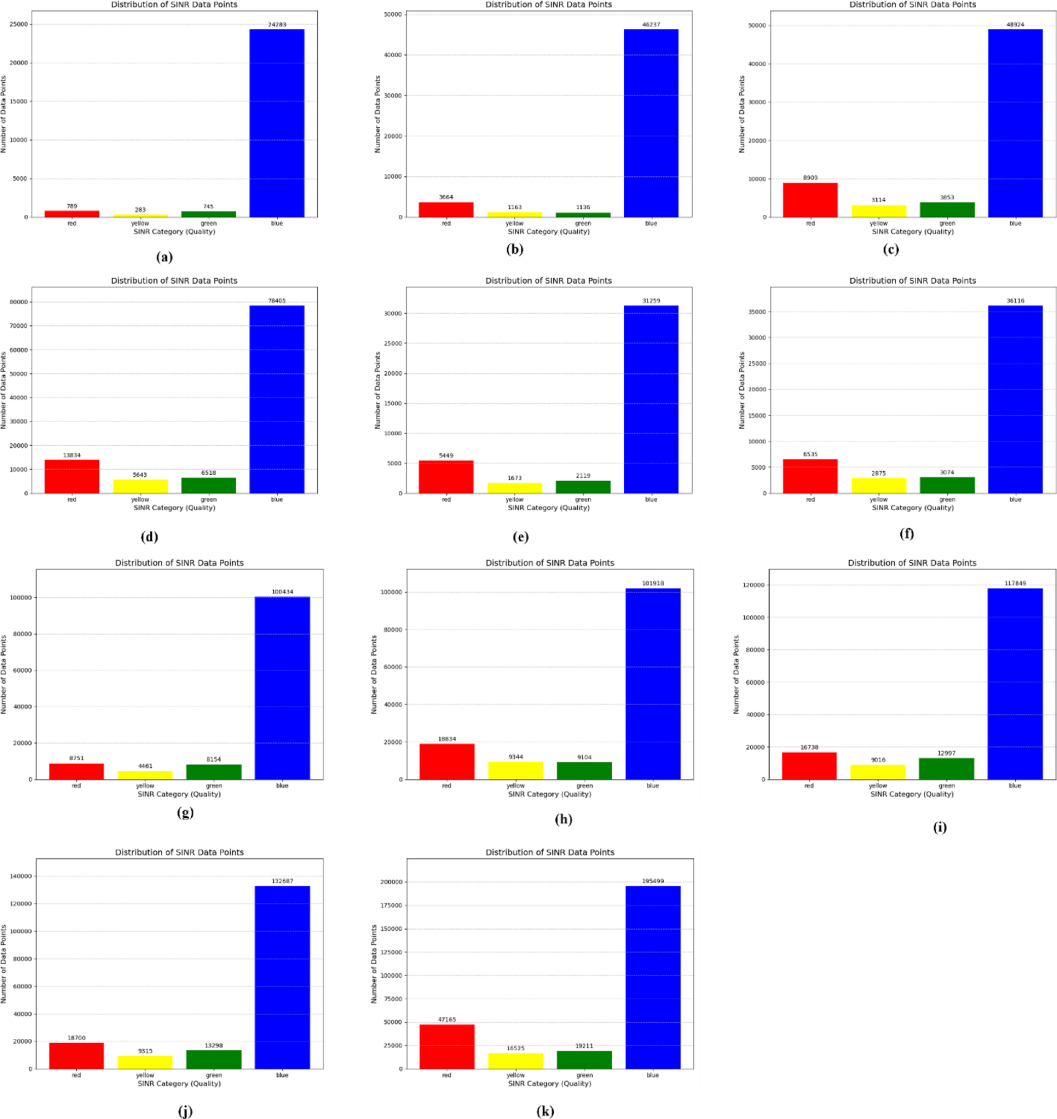
Number of SINR points at different level for BRKGA (**a**) 6eNodeBs, (**b**) 12eNodeBs, (**c**) 18eNodeBs, (**d**) 24eNodeBs, (**e**) 30eNodeBs, (**f**) 36eNodeBs, (**g**) 42eNodeBs, (**h**) 48eNodeBs, (**i**) 54eNodeBs, (**j**) 60eNodeBs, (**k**) 96eNodeBs).

 In addition, Fig. 10 presents the UE trajectories for the BRKGA-based PCI allocation, followed by the corresponding SINR distributions for different network sizes. The frequency of each SINR class is summarized in the accompanying tables. Overall, the results are consistent with those obtained using DSATUR. Although certain eNodeB configurations exhibit a slight reduction in the highest SINR class, the same performance trends observed for DSATUR remain valid. A direct performance comparison between BRKGA and DSATUR is provided in the next subsection. Figure 11 illustrates the SINR improvement achieved by BRKGA relative to conventional PCI assignment, while Tables 9 and 10 confirm the overall SINR enhancement across the simulated scenarios.

**Table 9:** Number of data points at different SINR points for BRKGA.

SINR Category	SINR Range (dB)	Number of Data Points										
		6e NB	12eNB	18e NB	24e NB	30e NB	36e NB	42e NB	48e NB	54e NB	60e NB	96e NB
Red	< 5	789	945	8909	13,834	5449	6535	8751	18,834	16,738	18,700	47,165
Yellow	5: 10	283	528	3114	5643	1673	2875	4461	9344	9016	9315	16,525
Green	10: 20	745	457	3853	6518	2119	3074	8154	9104	12,997	13,298	19,211
Blue	>= 20	24,283	14,270	48,924	78,405	31,259	36,116	100,434	101,918	117,849	132,687	195,499

**Table 10:** Relative frequency at different SINR points for BRKGA.

SINR Category	SINR Range (dB)	Relative Frequency (%) BRKGA										
		6eNodeB	12eNodeB	18eNodeB	24eNodeB	30eNodeB	36eNodeB	42eNodeB	48eNodeB	54eNodeB	60eNodeB	96eNodeB
Red	< 5	3.02	5.83	13.75	13.25	13.45	13.45	7.18	13.53	10.69	10.75	16.94
Yellow	5:10	1.08	3.26	4.81	5.41	4.13	5.92	3.66	6.71	5.76	5.35	5.94
Green	10:20	2.85	2.82	5.95	6.24	5.23	6.33	6.69	6.54	8.3	7.64	6.9
Blue	>= 20	93.04	88.09	75.5	75.1	77.18	74.31	82.46	73.22	75.25	76.26	70.22

**Fig. 12:**
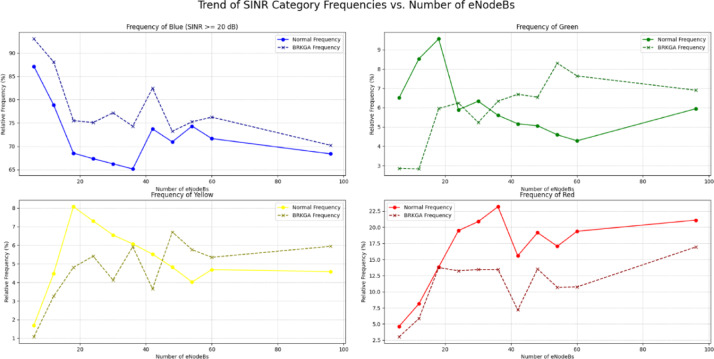
SINR comparison (BRKGA vs. normal iteration).

**Fig. 13:**
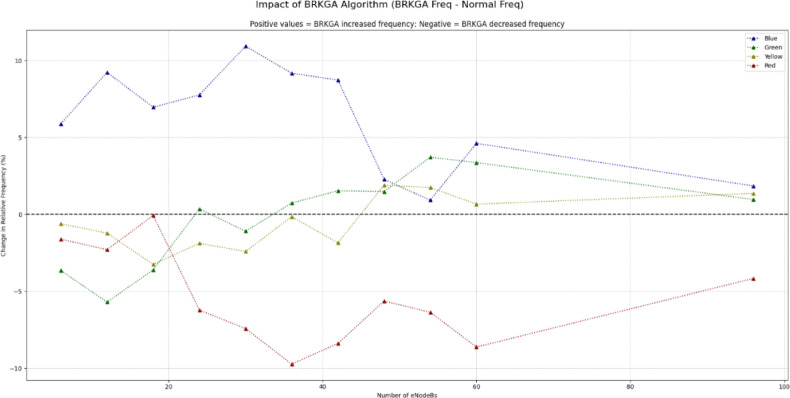
**Difference** between BRKGA and normal run (SINR).

Furthermore, Figs. 12 and 13 perform a comprehensive overview of the performance of BRKGA in enhancing SINR with respect to the number of nodes. As can be seen, BRKGA provides SINR improvement along all the network configurations(sizes) especially in blue SINR class. It also provides a huge reduction in red SINR class, with a slight increase in yellow and green classes.

### ILP results

**Fig. 14:**
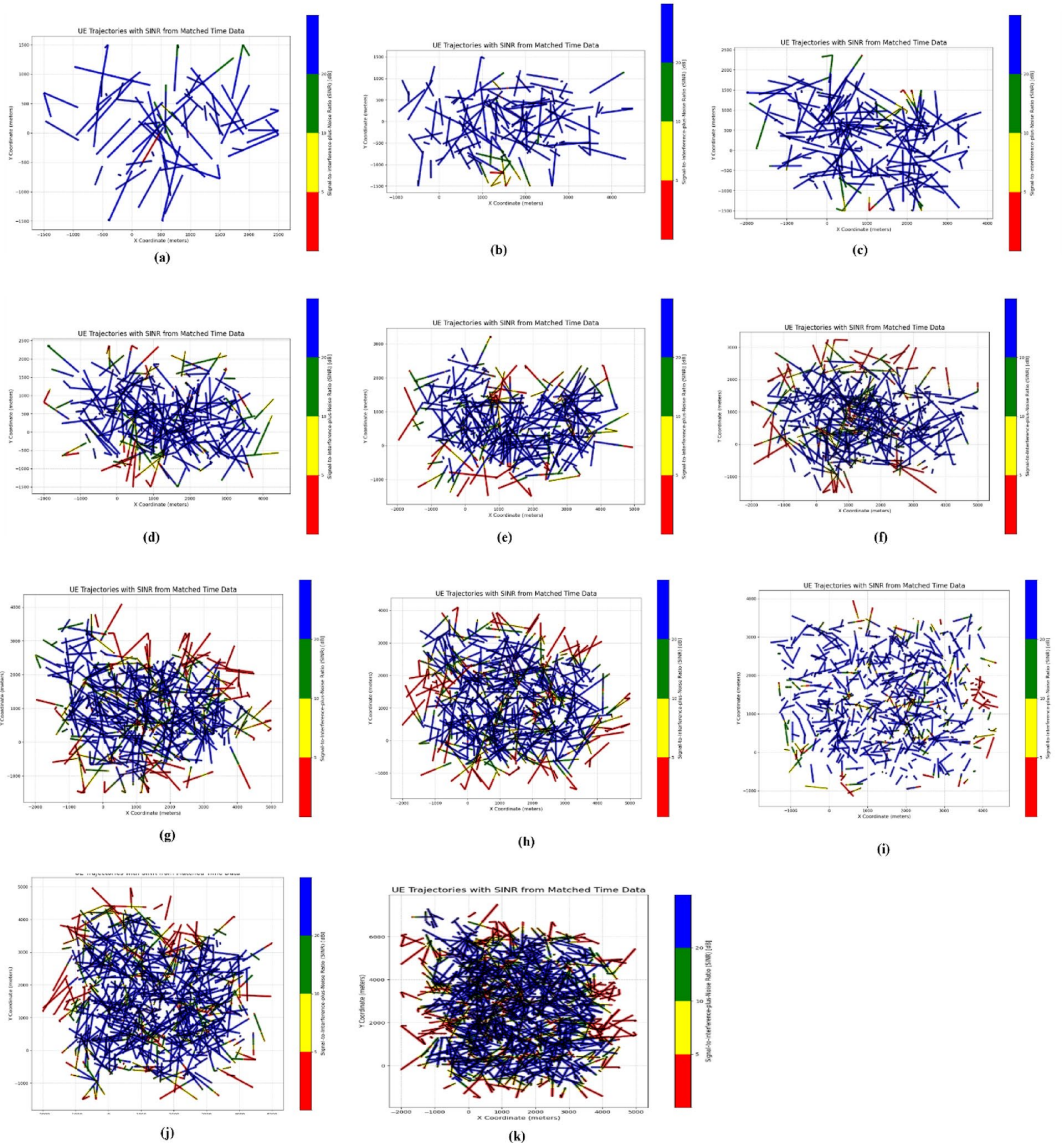
Random movement of UE trajectories at different number of eNodeBs using ILP clustering ((**a**) 6eNodeBs, (**b**) 12eNodeBs, (**c**) 18eNodeBs, (d) 24eNodeBs, (e) 30eNodeBs, (f) 36eNodeBs, (g) 42eNodeBs, (h) 48eNodeBs, (**i**) 54eNodeBs, (**j**) 60eNodeBs, (**k**) 96eNodeBs).

**Fig. 15:**
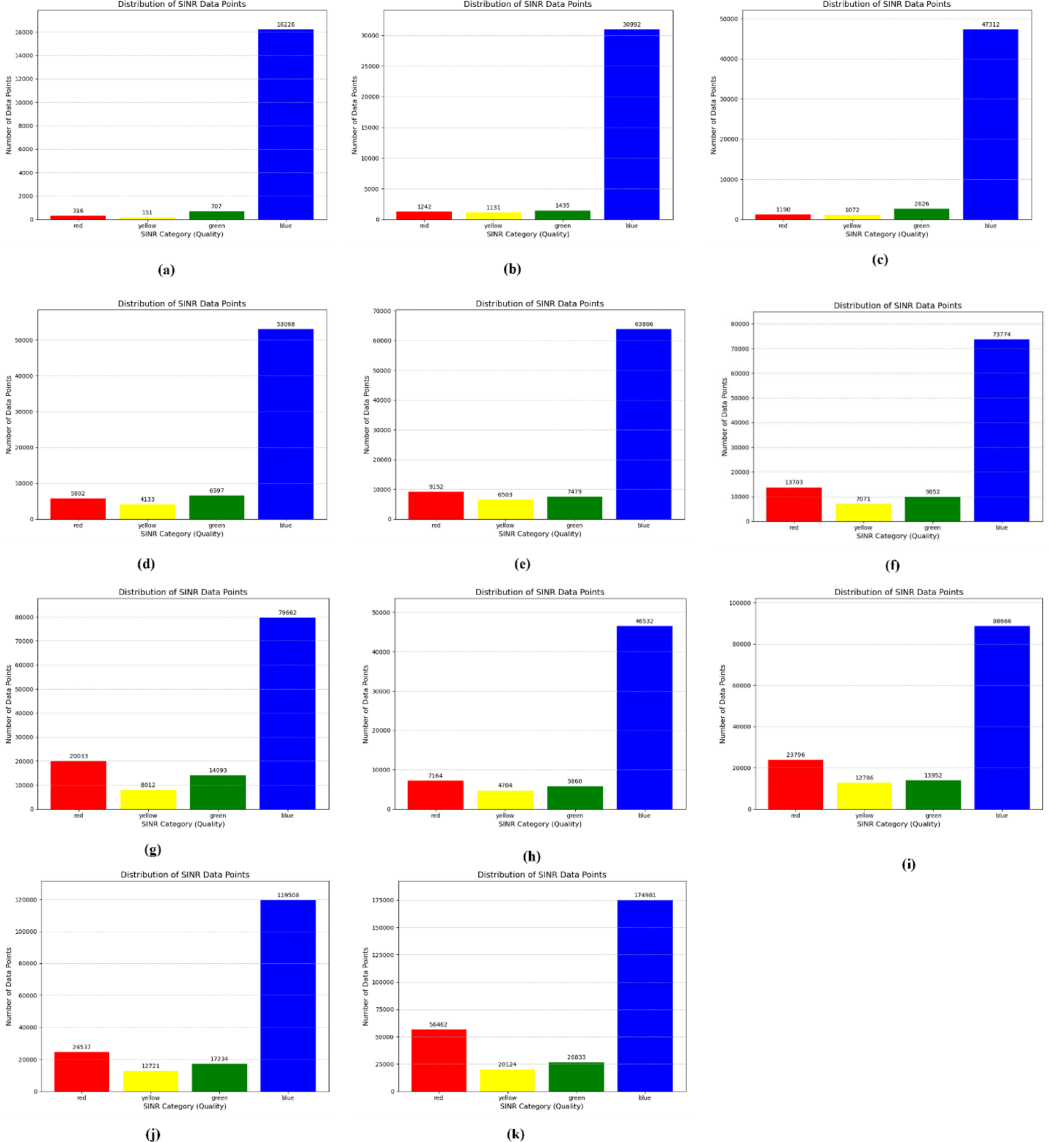
Number of SINR points at different level for ILP clustering (**a**) 6eNodeBs, (**b**) 12eNodeBs, (**c**) 18eNodeBs, (**d**) 24eNodeBs, (**e**) 30eNodeBs, (**f**) 36eNodeBs, (**g**) 42eNodeBs, (**h**) 48eNodeBs, (**i**) 54eNodeBs, (**j**) 60eNodeBs, (**k**) 96eNodeBs).

**Fig. 16:**
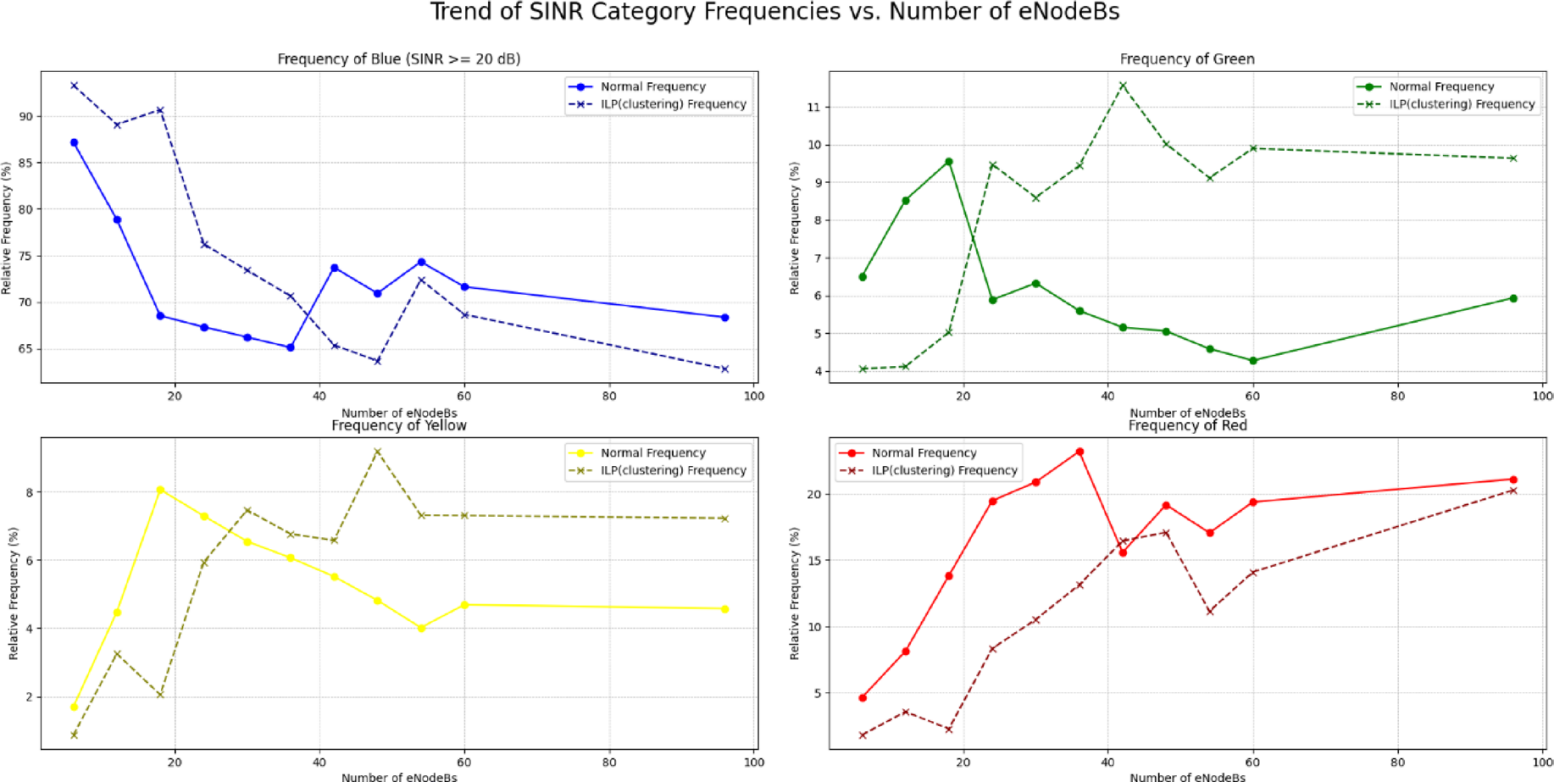
SINR comparison (ILP(Clustering) versus normal iteration.

**Fig. 17:**
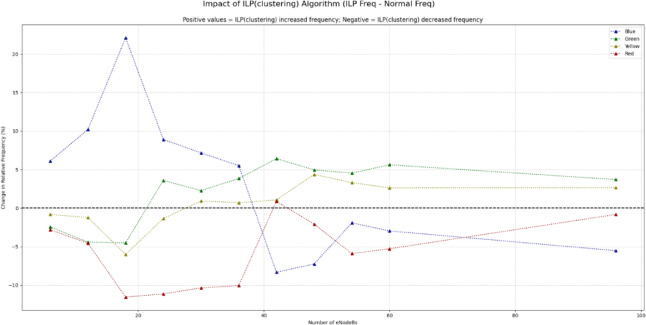
SINR Difference between ILP(Clustering) at different eNodeBs.

 As with DSATUR, we first present the UE trajectories in Fig. 14, followed by the SINR distribution across different numbers of eNodeBs in Fig. 15. Tables 11 and 12 summarize the frequency of each SINR class, showing results similar to those obtained with ILP. While some eNodeB configurations lead to a slight reduction in the blue SINR points, the same observations noted for DSATUR still apply. Figure 16 compares the normal PCI iteration with PCI allocation using ILP, highlighting the improvements in SINR distribution. The performance of ILP in Fig. 17 provides improvement in SINR if the network is so small. At larger networks (larger than 40 nodes) ILP provides less SINR. However, it provides reductions in the red SINR class almost all the time. This comes at the expense of yellow and green classes increasing.

**Table 11:** Number of data points at different SINR points for ILP clustering.

SINR Category	SINR Range (dB)	Number of Data Points										
		6eNodeB	12eNodeB	18eNodeB	24eNodeB	30eNodeB	36eNodeB	42eNodeB	48eNodeB	54eNodeB	60eNodeB	96eNodeB
Red	< 5	316	1242	1190	5802	9152	13,703	20,033	23,796	7164	24,537	20.28
Yellow	5: 10	151	1131	1072	4133	6503	7071	8012	12,786	4704	12,721	7.23
Green	10: 20	707	1435	2626	6597	7479	9852	14,093	13,952	5860	17,234	9.64
Blue	>= 20	16,226	30,992	47,312	53,068	63,866	73,774	79,662	88,666	46,532	119,508	62.85

**Table 12:** Relative frequency at different SINR points for ILP clustering.

SINR Category	SINR Range (dB)	Relative Frequency ILP (clustering)										
		6eNodeB	12eNodeB	18eNodeB	24eNodeB	30eNodeB	36eNodeB	42eNodeB	48eNodeB	54eNodeB	60eNodeB	96eNodeB
Red	< 5	1.82	3.57	2.28	8.34	10.52	13.13	16.45	17.09	11.15	14.1	21.11
Yellow	5:10	0.87	3.25	2.05	5.94	7.47	6.77	6.58	9.19	7.32	7.31	4.58
Green	10:20	4.06	4.12	5.03	9.48	8.6	9.44	11.57	10.02	9.12	9.9	5.94
Blue	>= 20	93.25	89.06	90.64	76.25	73.41	70.66	65.4	63.7	72.41	68.68	68.38

Figure 17 shows the difference between SINR class percentages, between the Normal and the optimized runs with ILP (clustering). The portion where the blue curve is above zero is where the algorithm provides an enhancement in the SINR.

### Comparative analysis and discussion

#### Comparison of SINR enhancement

In scenarios with a low density of network nodes, the primary goal is to maximize coverage quality with limited resources. An evaluation of the algorithms under these conditions reveals a clear performance leader. The figures illustrate that for small-scale deployments, the DSATUR algorithm provides the most substantial improvement in user experience. At a deployment of 6 eNodeBs, DSATUR increases the share of users in the highest SINR category (blue, >= 20 dB) by approximately 9.8% over the baseline—a significant margin. For comparison, ILP (clustering) offers a respectable 6.1% enhancement, while BRKGA provides a 5.9% boost. Furthermore, DSATUR excels at mitigating poor signal quality; it reduces the frequency of the worst SINR category (red, < 5 dB) from 4.64% down to just 1.4%, a far greater reduction than the other algorithms. This trend continues for 12 and 18-node networks, establishing DSATUR as the most effective algorithm for smaller, less complex network deployments. As the network grows in size and complexity, the interactions between nodes become more intricate, and the challenge of interference management shifts. In these higher-density scenarios, an algorithm’s scalability becomes its most critical attribute. While DSATUR performed best at lower node counts, its relative advantage diminishes as the network scales.

**Fig. 18:**
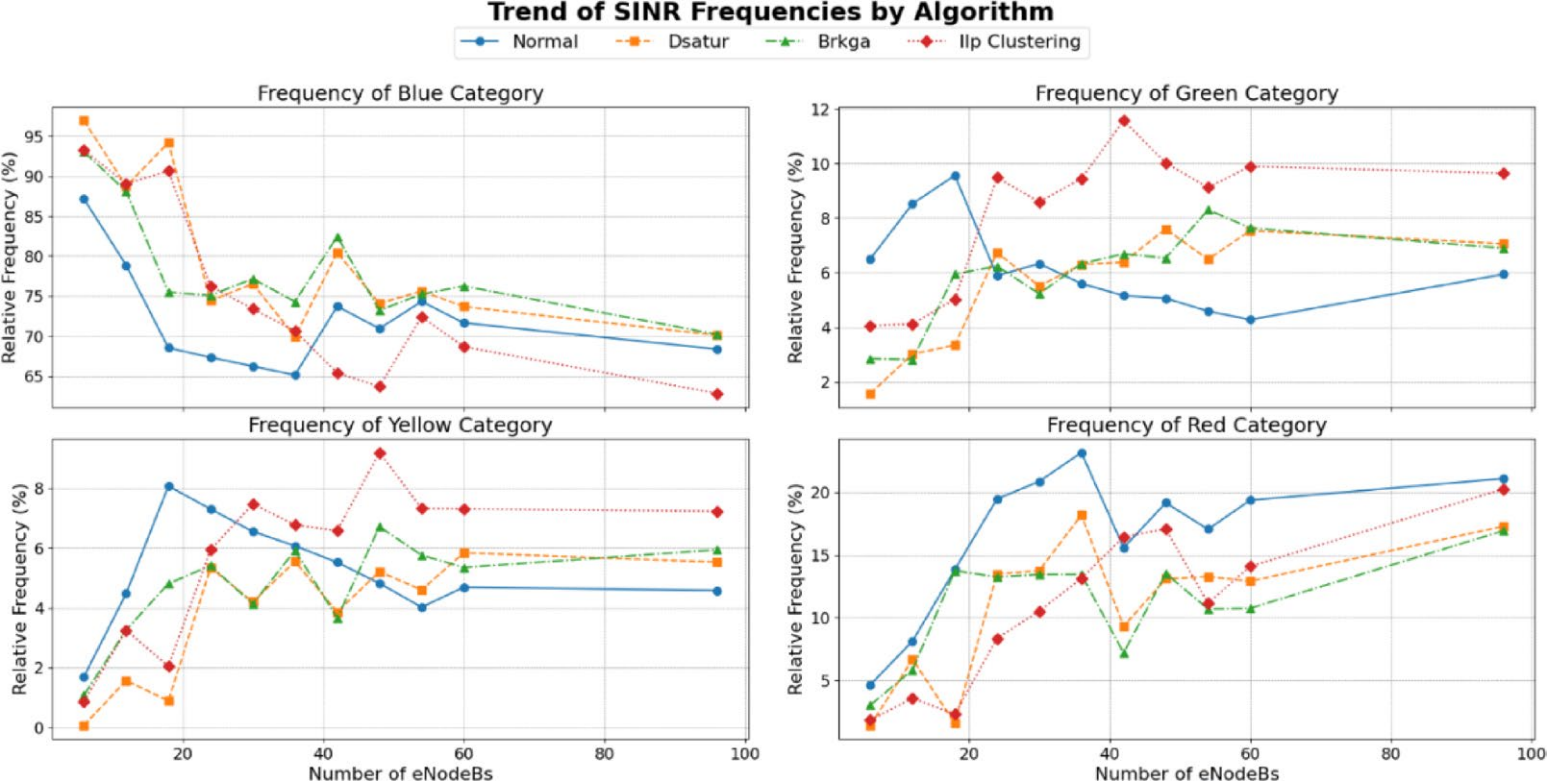
Relations between number of eNodeBs and relative frequency at different SINR levels for BRKGA, DSATUR, ILP (Clustering).

**Fig. 19:**
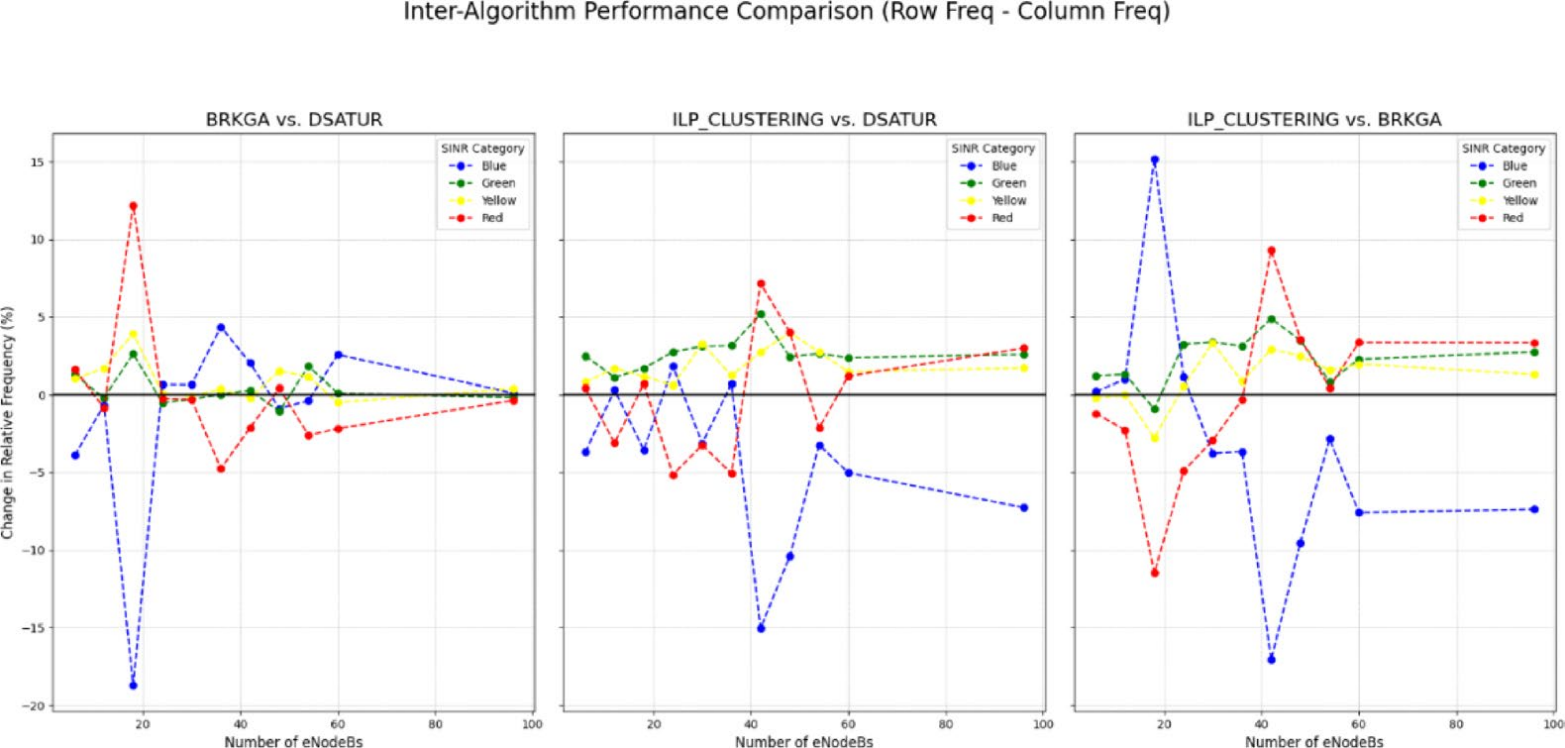
Comparison between BRKGA, DSATUR and ILP (Clustering) to show SINR improvements at different eNodeBs.

As in Figs. 18 and 19, the data indicates that the BRKGA algorithm is exceptionally well-suited for these more complex environments. At a 36 eNodeB deployment, BRKGA delivers the highest improvement to the blue SINR category, with an increase of 9.2% over the baseline, surpassing both ILP (clustering) (5.5%) and DSATUR (4.8%). At the largest simulated scale of 96 eNodeBs, both BRKGA and DSATUR provide a modest improvement of around 1.8% in the blue category. However, a critical finding appears with the ILP (clustering) algorithm. In the 96 eNodeB scenario, its performance degrades significantly, resulting in a 5.5% decrease in the highest SINR category compared to the baseline. This suggests that while effective at smaller scales, this implementation of ILP does not scale well to larger, denser network deployments and become counterproductive. To provide a quantitative basis for our comparison, we will focus on the Net Improvement for the high-quality blue SINR category (> = 20 dB). This metric is calculated by subtracting the baseline “Normal” frequency from the algorithm’s frequency at each eNodeB count.

- Positive Value: Indicates percentage point improvement over the baseline.
- Negative Value: Indicates a performance degradation compared to the baseline.

We will analyze the average net improvement across different network densities and calculate the overall performance slope to measure scalability.

**a. Low-Density Network Performance (6**,** 12**,** & 18 eNodeBs)**.

This analysis focuses on scenarios with fewer nodes, where initial deployment quality is paramount. The data clearly quantifies DSATUR’s dominance in low-density deployments. It achieves the highest average improvement of + 15.11%, significantly outperforming the other two algorithms. While ILP (clustering) is also a strong performer in this range with an average improvement of + 12.80%, it is DSATUR’s remarkable + 25.65% peak improvement at 18 eNodeBs that establishes it as the superior choice for small-scale networks. BRKGA is effective but provides roughly half the average benefit of DSATUR in these scenarios. Table 13 summarizes that.

**Table 13:** Statical analysis of SINR improvement for BRKGA, DSATUR and ILP (clustering) at small networks.

Algorithm	Net Improvement at 6 eNodeBs (%)	Net Improvement at 12 eNodeBs (%)	Net Improvement at 18 eNodeBs (%)	Average Low-Density Improvement (%)
DSATUR	+ 9.79	+ 9.89	+ 25.65	+ 15.11
ILP (clustering)	+ 6.10	+ 10.20	+ 22.10	+ 12.80
BRKGA	+ 5.89	+ 9.23	+ 7.00	+ 7.37

**b. High-Density Network Performance (36**,** 48**,** 60**,** & 96 eNodeBs)**.

This analysis inspects performance in larger, more complex networks where scalability and interference management are critical as in Table 14.

**Table 14:** Statical analysis of SINR improvement for BRKGA, DSATUR and ILP (clustering) at large networks.

Algorithm	Net Improvement at 36 eNodeBs (%)	Net Improvement at 96 eNodeBs (%)	Average High-Density Improvement (%)
BRKGA	+ 9.17	+ 1.84	+ 4.54
DSATUR	+ 4.81	+ 1.73	+ 2.20
ILP(clustering)	+ 5.52	−5.53	−0.19

In high-density scenarios, the performance dynamics shift dramatically. BRKGA emerges as the most robust algorithm, delivering the highest average improvement of + 4.54%. It also provides the best peak performance in this range, with a + 9.17% improvement at 36 eNodeBs. Most notably, the statistical analysis reveals the severe scalability problem of ILP(clustering). Its performance average becomes negative (−0.19%), driven by a significant 5.53% degradation at 96 eNodeBs. This confirms that what works well in a small network can become detrimental in a large one. DSATUR remains beneficial but its average improvement drops to + 2.20%, less than half of BRKGA’s.

c. Scalability Analysis: Performance Trend Slope.

As in Table 15, to statistically measure scalability, we can calculate the slope of the performance trendline for the Net Improvement across all eNodeB counts (6 to 96). A steeper negative slope indicates that the algorithm’s relative advantage decreases more rapidly as the network grows.

**Table 15:** Comparison of the scalability of the algorithms (Blue SINR class).

Algorithm	Performance Trend Slope (Improvement % per eNodeB)	Interpretation
BRKGA	−0.03	Highly Stable: Very little performance decay as the network scales.
DSATUR	−0.16	Moderate Scalability: Strong initial performance that declines steadily.
ILP (clustering)	−0.20	Poor Scalability: Strong initial performance that degrades rapidly.

This analysis provides the clearest picture of algorithmic suitability for long-term growth. BRKGA’s near-zero slope of −0.03 proves it is the most scalable and reliable algorithm. Its performance is remarkably consistent regardless of network size. Conversely, ILP (clustering) has the steepest negative slope (−0.20), statistically confirming that its benefits are quickly lost and eventually turn into a net negative as the network expands. DSATUR sits in the middle, confirming it is a strong choice for initial deployments but less ideal for networks expected to undergo significant expansion compared to BRKGA.

**d. Scalability Analysis for Red Class (SINR < 5 dB)**.

Table 16 analyzes the algorithms’ ability to reduce the frequency of the worst-performing signals as the network grows.

**Table 16:** Comparison of the scalability of the algorithms (RED SINR class).

Algorithm	Performance Trend Slope (% Change per eNodeB)	Interpretation
DSATUR	+ 0.16	Decreasing Effectiveness: Initially excellent at reducing red-class signals, but its effectiveness sharply diminishes, and it becomes less helpful than the baseline in larger networks.
BRKGA	−0.01	Highly Stable: Maintains a consistent and beneficial reduction of the worst signals across all network sizes.
ILP (clustering)	+ 0.16	Poor Scalability: Similar to DSATUR, its initial benefit quickly fades, and it becomes less effective at managing the worst signals as the network expands.

**e. Scalability Analysis for Yellow Class (SINR 5 to 10 dB)**.

Table 17 analyzes the algorithms’ effectiveness at reducing low-quality intermediate signals.

**Table 17:** Comparison of the scalability of the algorithms (Blue SINR class).

Algorithm	Performance Trend Slope (% Change per eNodeB)	Interpretation
DSATUR	−0.01	Slightly Increasing Effectiveness: Consistently reduces yellow-class signals, with a slight tendency to get better as the network grows.
BRKGA	+ 0.04	Slightly Decreasing Effectiveness: Offers a small benefit in smaller networks, but this advantage wanes and it slightly increases yellow-class signals in larger deployments compared to the baseline.
ILP (clustering)	+ 0.07	Poor Scalability: While beneficial in some small networks, it becomes progressively worse at managing this signal category, significantly increasing its frequency in larger networks.

**f. Scalability Analysis for Green Class (SINR 10 to 20 dB*)***.

Table 18 analyzes the algorithms’ ability to upgrade users from a “good” signal quality to an “excellent” one (the blue class). A negative slope indicates an increasing ability to clear this category.

**Table 18:** Comparison of the scalability of the algorithms (Blue SINR class).

Algorithm	Performance Trend Slope (% Change per eNodeB)	Interpretation
DSATUR	+ 0.01	Stable Impact: Consistently increases the frequency of green-class signals across all network sizes with a very stable impact.
ILP(clustering)	+ 0.02	Slightly Decreasing Effectiveness: Its ability to upgrade users from this category lessens slightly as the network grows larger.
BRKGA	−0.01	Stable Impact: it shows a consistent and stable ability to move users out of the green category and into the blue one.

**g. Coefficient of Variation (CV)**.

This metric measures the volatility or consistency of an algorithm’s performance relative to its average. A lower CV is highly desirable, as it indicates a more predictable and stable algorithm whose performance you can rely on regardless of network size (CV = (Standard Deviation/Mean) * 100%).

BRKGA is the clear winner in terms of consistency. With a CV of only 56.5%, its performance is far more stable and predictable than the others’. This is the statistical signature of a robust, scalable algorithm. DSATUR and ILP (clustering) have extremely high CVs (over 100%). This statistically proves that they are unreliable and highly situational. Their performance fluctuates dramatically depending on the network conditions (in this case, the number of eNodeBs). This quantifies the “rise and fall” pattern we observed visually. Table 19 summarizes the result.

**Table 19:** CV of BRKGA, DSATUR and ILP (Clustering.

Algorithm	Mean Net Improvement (%)	Standard Deviation	Coefficient of Variation (CV)	Performance Profile
BRKGA	4.58	2.59	56.5%	Most Stable & Predictable
ILP (clustering)	8.11	8.76	108.0%	High-Reward, High-Risk
DSATUR	8.00	8.87	110.8%	Most Volatile

**h. Paired T-Test**.

To rigorously compare the algorithms, a paired t-test is used. This test determines if the average difference in performance between two algorithms is statistically significant, meaning it is unlikely to have occurred by random chance. We analyze the Net Improvement in the blue SINR category across all 11 eNodeB counts for each algorithm pair. The critical output is the p-value. A p-value less than 0.05 indicates that we can be more than 95% confident that a real performance difference exists between the two algorithms being compared. Table 20 summarizes the result.

**Table 20:** Paired T-Test between BRKGA, DSATUR and ILP(Clustering).

Algorithm Pair	Mean Difference (%) (Positive value means the first algorithm was better on average)	Provided *p*-value	Is the Difference Significant?
DSATUR vs. ILP (clustering)	+ 4.42	0.015	Yes
BRKGA vs. ILP (clustering)	+ 1.00	0.228	No
DSATUR vs. BRKGA	+ 3.41	0.504	No

Based on this set of results, we can draw clear and distinct conclusions about the relative performance of the algorithms. DSATUR vs. ILP (clustering): This comparison yields the most important finding. With a p-value of 0.015, which is well below the 0.05 threshold, there is strong statistical evidence that DSATUR provides a significantly higher average improvement to the blue SINR category than ILP (clustering). The observed average advantage of + 4.42% for DSATUR is not a random fluke but a reliable and statistically significant performance gap. In addition, BRKGA vs. ILP (clustering): The p-value of 0.228 is considerably higher than 0.05. This indicates that there is no statistically significant difference between the average performance of BRKGA and ILP(clustering). While their performance behaviors are very different (one is stable, the other volatile), their overall average impact across the tested scenarios is statistically equivalent. Furthermore, DSATUR vs. BRKGA: Similarly, the p-value of 0.504 is very high, showing that there is no statistically significant difference in the average performance between DSATUR and BRKGA. Despite DSATUR having a higher mean improvement, the result is not consistent enough to claim a definitive statistical advantage over BRKGA.

#### Comparison of computational efficiency

**Fig. 20:**
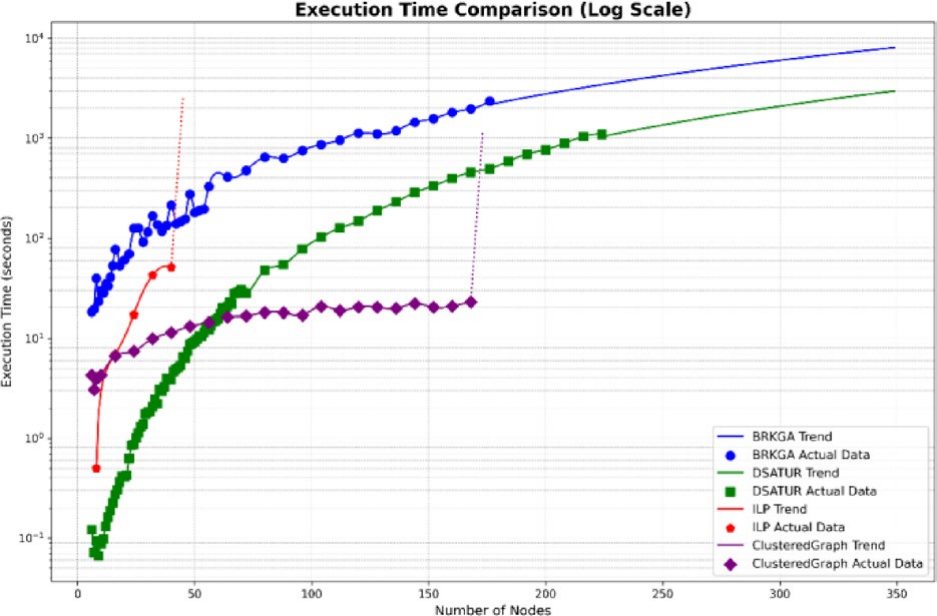
Computational run time comparison between BRKGA, ILP and DSATUR based C++.

**Fig. 21:**
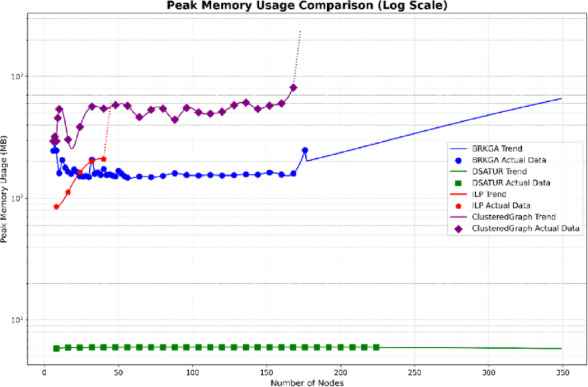
Memory requirement comparison between BRKGA, ILP and DSATUR based C++.

This section analyzes the computational efficiency of the three optimization algorithms: DSATUR, BRKGA, and ILP. DSATUR, implemented in C++, exhibits significantly faster run times and lower memory requirements compared to BRKGA and ILP. To reduce NS3 simulation time, random graphs representing networks were generated (6–300 nodes, sparse connectivity, maximum 7 edges per node). The number of top neighbors for each node was set to 20% of the total nodes. PCI allocation solutions were obtained from these graphs, and cubic spline interpolation (degree 3) was used to estimate additional data points. Run-time analysis shows that DSATUR is much faster, with a near-linear increase with network size, while BRKGA’s run-time grows faster. ILP fails for larger or highly connected graphs (around 48 nodes). An improved ILP version combined with clustering allows feasible solutions for larger networks, though performance depends on connectivity. Python implementations of all algorithms confirmed DSATUR’s clear run-time advantage, while ILP with clustering performs better on large networks when feasible. Figures 20 and 21 illustrate these comparisons, preparing for the next section on overall performance and deployment scenarios.

**Fig. 22:**
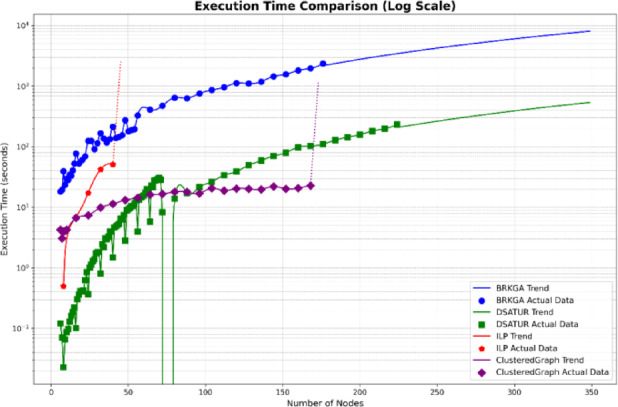
Computational run time comparison between BRKGA, ILP and DSATUR based C++.

**Fig. 23:**
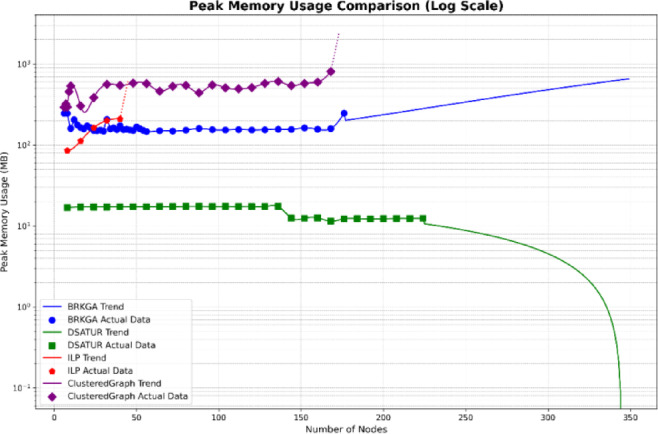
Memory requirement comparison between BRKGA, ILP and DSATUR based C++.

## Conclusion

This project tackled the persistent challenge of Physical Cell ID (PCI) allocation in cellular networks, where poor planning often causes interference and reduces user experience. To address this, three optimization algorithms—DSATUR, BRKGA, and ILP with clustering—were implemented and tested through extensive NS3 simulations, resulting in a practical, data-backed framework for improving SINR performance. The results show that all three algorithms outperform the baseline iterative allocation method, achieving roughly an 8% increase in high-quality SINR on average. However, their effectiveness varies based on network size and deployment goals. DSATUR excelled in small or rapid deployments, delivering the highest SINR improvements with extremely low computation time. Moreover, BRKGA proved the most scalable and dependable, maintaining consistent performance even in large networks. In contrast, ILP with clustering, although useful academically, lacked practicality due to high computational demands and poor scalability. Statistical validation reinforced these findings. A paired t-test confirmed DSATUR’s significant performance advantage over ILP. More importantly, Coefficient of Variation analysis showed that BRKGA is highly stable (CV of 56.5%), while DSATUR and ILP are highly inconsistent (CVs above 100%), highlighting the trade-off between peak performance and reliability. Rather than naming a single best algorithm, the study introduces a context-aware strategy for PCI optimization. A two-phase model is recommended: the first one is DSATUR for early-stage or small-area deployments where speed and immediate performance are crucial. And the last one is BRKGA for large or mature networks that require long-term stability and predictable outcomes. The study acknowledges limitations such as NS3’s fixed coupling of PCI and Cell-ID and the use of a constant ANR threshold. Future work should consider variable thresholds, 5G NR simulations, handover-weight network graphs, and uniform implementation of all algorithms in one high-performance language. In essence, this work offers operators a validated, adaptable framework for smarter PCI allocation. By selecting algorithms based on deployment context, operators can improve SINR, enhance user data rates, maintain network stability, and enable more efficient network management in the future. To sum up, this study offers a thorough, simulation-driven assessment of several PCI allocation algorithms under practical LTE/NR network conditions, including ILP, DSATUR, BRKGA, and clustering-based techniques. Our findings quantify decreases in PSS conflicts, show trade-offs between performance and complexity, and emphasize the significance of neighboring relationships driven by handover and RSRP-based adjacency. The suggested framework creates reliable baselines and a comparative basis for further work, despite its reliance on traditional optimization techniques. AI-based PCI prediction, extensions to 6G NR-V2X networks, and dynamic PCI reassignment in edge computing scenarios—which enables adaptive and real-time network optimization—are some possible future directions.

## Data Availability

The datasets generated and/or analyzed during the current study are available from the corresponding author Samar I. Farghaly on reasonable request.

## Code availability

The dataset is publicly available on GitHub: https://github.com/MarwanHelmy/Graduation-Project.
